# A Druggable Tumor Suppressor and Leukemic Stem Cell Marker

**DOI:** 10.1101/2025.10.16.682831

**Published:** 2025-12-22

**Authors:** Yi Pan, Xiaduo Meng, Chen Wang, Wenxuan Zhou, Yibo Chen, Richard D. Hammer, Hong Zheng, Gerhard Hildebrandt, Xunlei Kang

**Affiliations:** 1 Center for Precision Medicine, Department of Medicine, University of Missouri School of Medicine, Columbia, MO 65212, USA; 2 Ellis Fischel Cancer Center at MU Health Care, University of Missouri, Columbia, MO 65212, USA; 3 Division of Hematology and Oncology, Department of Medicine, University of Missouri School of Medicine, Columbia, MO 65212, USA; 4 Institute for Data Science and Informatics, University of Missouri, Columbia, MO, 65211 USA; 5 Department of Pathology and Anatomical Sciences, University of Missouri School of Medicine, Columbia, MO 65212, USA; 6 Division of Hematology/Oncology, Penn State University College of Medicine, Hershey, PA 17033, USA

## Abstract

Acute myeloid leukemia (AML) often enters remission after chemotherapy but frequently relapses due to chemotherapy-resistant leukemic stem cells (LSCs). Relapsed AML remains largely unresponsive to current therapies and carries a poor prognosis. We developed a large-language model (LLM) agent that incorporates multi-modal data to nominate druggable therapeutic targets for AML. We identified that higher expression of *AGTR2* (encoding AT2R) is associated with better chemotherapy response and longer survival. In functional studies of 68 primary human AML samples, we found that LSCs consistently lacked AT2R expression. Across both CD34-expressing and -non-expressing AML samples, AT2R expression positively correlated with CD34 and CD117 expression. In patient-derived xenograft (PDX) models using 21 primary AML samples, AT2R⁻ cells initiated leukemia, whereas AT2R⁺ cells failed to do so. AT2R⁻ cells gave rise to both AT2R⁺ and AT2R⁻ progeny, suggesting hierarchical differentiation. Following chemotherapy in AML PDX mice, bone marrow analysis showed a marked enrichment of AT2R⁻ cells and depletion of AT2R⁺ cells, indicating that AT2R⁻ cells drive minimal residual disease and relapse. These results support the role of AT2R absence as a marker of LSCs. We observed reduced AT2R expression in AML cells compared to healthy peripheral blood and bone marrow mononuclear cells, suggesting a tumor suppressor role. Whole-genome sequencing of AML patients revealed no functional mutations in *AGTR2*. However, 3D chromatin and epigenetic analyses uncovered frequent chromatin rearrangements involving *AGTR2* promoter–silencer interactions, indicating epigenetic silencing as a likely mechanism for AT2R downregulation in AML. To validate the tumor suppressor role of AT2R, we developed murine AML models driven by MLL-AF9 or AML1-ETO9a fusions with either *Agtr2* knockdown or enforced expression. *Agtr2* knockdown accelerated leukemogenesis, while enforced *Agtr2* expression delayed AML progression. In these models, enforced *Agtr2* expression reduced LSC frequency, impaired cell cycle progression, and decreased AML stemness, as confirmed by limiting dilution assays and analysis of LSC-enriched populations. Mechanistically, enforced *Agtr2* expression suppressed fatty acid metabolism – a key driver of AML stemness and growth – and inhibited downstream signaling pathways, including GSK3, PI3K/AKT, and Wnt/β-catenin. This led to reduced SREBF1 activity, confirmed by protein level changes and CUT&Tag assays. We tested buloxibutid (C21), a small-molecule AT2R agonist currently in phase II trials for idiopathic pulmonary fibrosis, in AML PDX models derived from 20 de novo and 6 relapsed AML samples. C21 significantly inhibited AML progression and enhanced the efficacy of chemotherapy, particularly in relapsed AML models.

## Introduction

Acute myeloid leukemia (AML) is characterized by frequent relapse despite initial therapeutic success. Although most patients achieve complete remission with induction chemotherapy, the majority eventually relapse and succumb to the disease^1^. Relapse is thought to arise from the persistence of leukemia stem cells (LSCs) with self-renewal capacity and intrinsic drug resistance^2^. Traditionally, LSCs in AML are enriched in a CD34^+^CD38^−^ phenotype, similar to primitive hematopoietic stem cells. However, not all AML cases follow this pattern; approximately 25–30% of AML patients present with blasts that have low or absent CD34 expression^3^. This biological heterogeneity underscores the need to identify additional functional LSC markers and develop a more precise definition of the LSC state. A deeper understanding of LSC regulation is therefore critical for designing strategies that prevent relapse and achieve durable remission.

Tumor-suppressor loss represents another hallmark of AML pathogenesis, yet it frequently occurs without recurrent coding mutations. Beyond canonical lesions in *TP53*, *WT1*, *NF1*, or biallelic *CEBPA*, many tumor-suppressive programs are silenced epigenetically through promoter hypermethylation, polycomb-mediated H3K27me3 deposition, or long-range chromatin interactions that connect distal silencers to key regulatory promoters^4^. Such repression is dynamic and potentially reversible, creating therapeutic entry points distinct from mutation repair. Despite these opportunities, few tumor-suppressive pathways in AML are currently actionable, and even fewer have been mechanistically linked to LSC maintenance or therapy resistance.

To address this gap, we employed a large language model (LLM)–driven discovery pipeline that systematically mined phenotypic, transcriptomic, and druggability datasets to uncover candidate genes with tumor-suppressive features in AML. This unbiased computational approach identified *AGTR2* (encoding AT2R) as a key regulator of leukemia biology. Our integrated analyses revealed that AT2R is transcriptionally silenced in AML – particularly within LSC-enriched compartments – and that its loss correlates with poor patient prognosis. Reactivation of AT2R suppressed AML development through disrupted SREBP1-driven fatty acid oxidation. These findings establish AT2R loss as a defining feature of chemoresistant LSCs and nominate AT2R reactivation as a tractable strategy to restore a tumor-suppressive axis in AML.

Buloxibutid (C21) is a first-in-class, selective AT2R agonist with clinical experience across multiple human trials, providing a robust safety foundation for therapeutic translation^5–7^. We therefore conducted a preclinical, phase II–like study using patient-derived xenograft (PDX) models to evaluate the anti-leukemic efficacy of C21 and to identify biomarkers of response and resistance in AML. This experimental design also enabled within-cohort testing of rational combination strategies and direct benchmarking against standard-of-care regimens to quantify additive or synergistic effects.

## Results

### An LLM agent for AML targets nomination

We built a tool-augmented LLM agent that nominates therapeutic targets directly from an AML cohort’s RNA-expression and phenotype data. Instead of directly nominating targets through survival stratification, given the confounding factors commonly associated with survival data, we used the ELN 2022 risk group and therapeutic response as endpoints to nominate targets associated with favorable outcomes^8^.

The agent first derives primary signals from the cohort: genes that show significant expression between low-risk/favorable response versus high-risk/adverse response were filtered out. To maximize druggability, we annotated protein type encoded by each gene and selected only cell-surface targets. For each shortlisted gene, the agent assembles orthogonal evidence and synthesizes a “target dossier” from curated datasets, including prior pharmacology (DGIdb/ChEMBL)^9,10^ and interaction context and strength (STRING partners)^11^. Cross-validation was then assessed through elastic-net Cox models for overall survival using the BEATAML dataset^12^ and CRISPR essentiality using DepMap^13^. Ranked coefficients are contextualized by pathway enrichment (Hallmarks/Reactome) to verify biological coherence (e.g., apoptosis, cell-cycle, chromatin). This yields a shortlist of genes with prognostic or predictive signal. To contextualize each gene’s therapeutic novelty, we implemented an AI-assisted literature synthesis step. For each gene, PubMed abstracts and clinical trial records were retrieved and filtered using relevance and recency criteria. The top abstracts were then ranked using a hybrid BM25+ embedding model and provided, along with trial metadata, to a large language model (LLM). The LLM generated a structured summary describing the gene’s oncogenic role, therapeutic context, and supporting references, producing a concise, interpretable brief that complements quantitative novelty measures. We then apply an explicit human-genetics safety triage: gnomAD constraint (LOEUF/pLI)^14^ as a haploinsufficiency proxy; GTEx v8^15^ median TPM to penalize broad or high expression in critical tissues (brain, heart, liver, kidney, lung, blood); and breadth of cis-eQTLs across tissues. These components are combined into a composite priority with a safety penalty, favoring lineage-selective, druggable, and lower predicted on-target-toxicity candidates. The final output is a ranked target list and per-gene dossiers with transparent, traceable evidence. In our AML cohort, top-ranked genes showed convergence of cohort signal, essentiality, and pathway coherence; several understudied genes (*AGTR2*, *NTRK1*, *LAG3*, *MPL*, *TERT*) retained high priority after safety filtering, highlighting actionable and novel avenues for experimental validation.

### Absence of AT2R marks chemo-resistant LSCs in AML patients

**Fig. 2:**
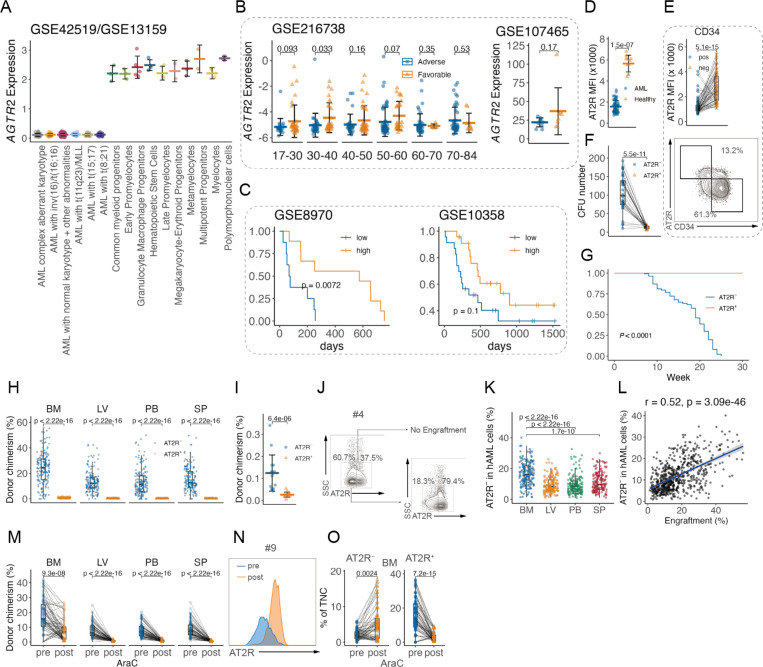
Absence of AT2R marks chemo-resistant LSCs in AML patients A, Normalized transcriptomic expression of *AGTR2* for AML cytogenetic subtypes and for normal hematopoietic/myeloid populations in GSE42519 and GSE13159 (AML complex aberrant karyotype, n = 48; AML with inv(16)/t(16;16), n = 28; AML with normal karyotype + other abnormalities, n = 351; AML with t(11q23)/MLL, n = 38; AML with t(15;17), n = 37; AML with t(8;21), n = 40; Common myeloid progenitors, n = 3; Early Promyelocytes, n = 3; Granulocyte Macrophage Progenitors, n = 5; Hematopoietic Stem Cells, n = 4; Late Promyelocytes, n = 3; Megakaryocyte-Erythroid Progenitors, n = 2; Metamyelocytes, n = 3; Multipotent Progenitors, n = 2; Myelocytes, n = 2; Polymorphonuclear cells, n = 3). *P* < 0.0001 in all comparisons of AML vs healthy cells. Wilcoxon rank-sum test. B, Normalized transcriptomic expression of *AGTR2* for AML with adverse or favorable outcomes stratified by age in GSE216738 (17–30 Adverse, n = 22; 17–30 Favorable, n = 34; 30–40 Adverse, n = 36; 30–40 Favorable, n = 29; 40–50 Adverse, n = 34; 40–50 Favorable, n = 39; 50–60 Adverse, n = 54; 50–60 Favorable, n = 29; 60–70 Adverse, n = 45; 60–70 Favorable, n = 7; 70–84 Adverse, n = 46; 70–84 Favorable, n = 9) or not stratified in GSE107465 (Adverse, n = 10; Favorable, n = 8). Wilcoxon rank-sum test. C, Survival curve of AML patients stratified by transcriptomic expression of *AGTR2* in GSE8970 (low, n = 0, high, n = 8) and GSE10358 (n = 23). Log-rank test. D, Flow cytometry analysis of the comparison of AT2R MFI in human AML cells (n = 62 cases of AML) and healthy control cord blood (n = 11 cases of healthy individuals). E, Flow cytometry analysis of the comparison of AT2R MFI in CD34^**+**^ vs CD34^**−**^ fractions of human AML cells (top, n = 62 cases of AML) and representative plot (bottom). median with IQR. F, Quantification of in vitro colony-forming unit (CFU) assay (n = 32 cases of AML: 1, 2, 3, 4, 5,, 6, 7, 8, 9, 10, 11, 12, 13, 14, 15, 16, 17, 18, 19, 20, 21, 22, 23, 24, 25, 26, 27, 28, 29, 30,, 31, 32). Connected dots depict colony-forming units of AT2R^**+**^ or AT2R^**−**^ fractions from one sample. G, Survival curve of human AML-transplanted mice (n = 21 cases of AML. Transplanted mice: No. 1 AT2R^**−**^, n = 6; No. 1 AT2R^**+**^, n = 5; No. 4 AT2R^**−**^, n = 6; No. 4 AT2R^**+**^, n = 5; No. 7 AT2R^**−**^, n = 7; No. 7 AT2R^**+**^, n = 5; No. 10 AT2R^**−**^, n = 6; No. 10 AT2R^**+**^, n = 5; No. 18 AT2R^**−**^, n = 6; No. 18 AT2R^**+**^, n = 5; No. 22 AT2R^**−**^, n = 8; No. 22 AT2R^**+**^, n = 5; No. 24 AT2R^**−**^, n = 7; No. 24 AT2R^**+**^, n = 5; No. 25 AT2R^**−**^, n = 5; No. 25 AT2R^**+**^, n = 5; No. 26 AT2R^**−**^, n = 6; No. 26 AT2R^**+**^, n = 5; No. 29 AT2R^**−**^, n = 8; No. 29 AT2R^**+**^, n = 5; No. 38 AT2R^**−**^, n = 8; No. 38 AT2R^**+**^, n = 5; No. 40 AT2R^**−**^, n = 6; No. 40 AT2R^**+**^, n = 5; No. 49 AT2R^**−**^, n = 5; No. 49 AT2R^**+**^, n = 5; No. 52 AT2R^**−**^, n = 8; No. 52 AT2R^**+**^, n = 5; No. 54 AT2R^**−**^, n = 5; No. 54 AT2R^**+**^, n = 5; No. 55 AT2R^**−**^, n = 8; No. 55 AT2R^**+**^, n = 5; No. 58 AT2R^**−**^, n = 6; No. 58 AT2R^**+**^, n = 5; No. 62 AT2R^**−**^, n = 7; No. 62 AT2R^**+**^, n = 5; No. 65 AT2R^**−**^, n = 6; No. 65 AT2R^**+**^, n = 5; No. 69 AT2R^**−**^, n = 7; No. 69 AT2R^**+**^, n = 5; No. 71 AT2R^**−**^, n = 6; No. 71 AT2R^**+**^, n = 5). Log-rank test. H, Flow cytometry analysis of long-term engraftment in NSG mice (n = 21 cases of AML: 1, 4, 7, 10, 18, 22, 24, 25, 26, 29, 38, 40, 49, 52, 54, 55, 58, 62, 65, 69, 71. n = 8 mice). median with IQR. I, Percentage of human AML cells that home to the bone marrow (n = 5 cases of AML: 1, 4, 26, 38, 40. n = 3 mice). J and K, Representative plots (J) and summarized results (K) of AT2R^**−**^ AML cells from engrafted mice (post-transplantation) (n = 21 cases of AML: 1, 4, 7, 10, 18, 22, 24, 25, 26, 29, 38, 40, 49, 52, 54, 55, 58, 62, 65, 69, 71. n = 8 mice). L, Linear regression analysis of correlation between percentage of AT2R^**−**^ AML cells and human AML chimerism in transplanted NSG mice (n = 21 cases of AML: 1, 4, 7, 10, 18, 22, 24, 25, 26, 29, 38, 40, 49, 52, 54, 55, 58, 62, 65, 69, 71. n = 8 mice). M to O, Mice engrafted with AML cells were treated with AraC (50 mg/kg daily for 5 days), and percentages of AML cells (M) or AT2R^**−**^ and AT2R^**+**^ AML cells of total nucleated cells (TNC) (O) were analyzed before and after treatment (n = 9 cases of AML: 4, 10, 18, 24, 38, 49, 52, 55, 65. n = 8 mice). Connected dots depict percentages before and after treatment in one mouse. Representative plot is shown (N). Unless noted, each dot represents an individual sample or mouse, horizontal bars and whiskers denote mean and SD, and *P* values are annotated and calculated using student’s t test.

We analyzed the expression of the top-ranked gene, *AGTR2* (encoding AT2R)m, in multiple public datasets and found that *AGTR2* shows higher expression levels in AML across different subtypes compared with hematopoietic stem and progenitor cells (HSPCs) (Fig. 2A). In AML patients, higher expression of *AGTR2* is associated with favorable chemotherapy response (Fig. 2B) and longer survival (Fig. 2C). We then screened 62 primary human AML samples and 11 peripheral blood mononuclear cells (PBMCs) from healthy controls for expression of AT2R. We found an overall lower expression of *AGTR2* in AML samples (Fig. 2D), accompanied by considerable heterogeneity in individual AML samples (Fig. S1A). Comparison of AT2R^+^ and AT2R^−^ fractions from individual AML samples found that AT2R^−^ fractions are significantly enriched in CD34^+^ LSC-enriched populations (Fig. 2E). In functional assays, AT2R^−^ fractions showed enhanced in vitro clonogenicity (Fig. 2F) and a highly specific ability to repopulate immunodeficient (NSG, NOD.Cg-*Prkdc*^*scid*^ *Il2rg*^*tm1Wjl*^/SzJ) mice (n = 21 AML samples; AT2R^−^ cells, 155 engrafted out of 168 transplanted mice (92%); AT2R^+^ cells, 0 engrafted out of 168 transplanted mice (0%); Fig. 2G and H). Mice transplanted with AT2R^−^, but not with corresponding AT2R^+^, AML cells showed leukemic infiltration of the hematopoietic organs as well as reduced overall survival (Fig. 2G and H; Fig. S1B and C). In homing assay, the ability of AT2R^+^ AML cells to home to the bone marrow (BM) was significantly reduced (Fig. 2I). Such observation is not biased by anti-AT2R antibody staining (Fig. S1D). Interestingly, AT2R^−^ cells gave rise to both AT2R^−^ and AT2R^+^ progeny in engrafted mice (Fig. 2J and K). AT2R^−^ cells are enriched in the BM (Fig. 2K; Fig. S1E) and show positive correlation with AML engraftment (Fig. 2L).

In mice engrafted with AML cells, in vivo treatment with chemotherapy (AraC, cytarabine) reduced overall AML infiltration (Fig. 2M; Fig. S1F) and AT2R^+^ AML cells, but not AT2R^−^ (Fig. 2N and O; Fig. 2G to J). In fact, AML engrafted mice show increased AT2R^−^ fraction of total nucleated cells (TNCs), likely due to the reduced overall cellularity in the BM while persistent AT2R^−^ cells, indicating that AT2R^−^ cells are resistant to chemotherapy.

### Absence of AT2R marks LSCs in both CD34-expression and -non-expressing AML

**Fig. 3:**
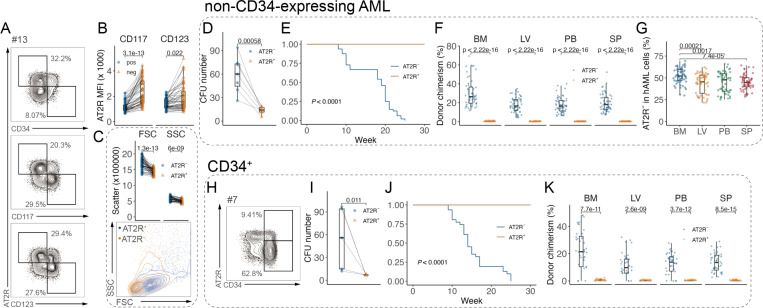
Absence of AT2R marks LSCs in both CD34-expression and -non-expressing AML A, Representative plots showing AT2R expression among CD34, CD117, and CD123 in a non-CD34-expressing AML sample (No. 4). B, Flow cytometry analysis of the comparison of AT2R MFI in CD117^**+**^ vs CD117^**−**^ or CD123^**+**^ vs CD123^**−**^ fractions of human AML cells (n = 62 cases of AML). C, Flow cytometry analysis of the comparison of FSC/SSC in AT2R^**+**^ vs AT2R^**−**^ fractions of human AML cells (top, n = 62 cases of AML) and representative plot (bottom). D, Quantification of in vitro CFU assay of non-CD34-expressing AML (n = 7 cases of AML: 13, 37, 42, 44, 51, 56, 63). Connected dots depict colony-forming units of AT2R^**+**^ or AT2R^**−**^ fractions from one sample. E, Survival curve of non-CD34-expressing human AML-transplanted mice (n = 7 cases of AML. Transplanted mice: No. 13 AT2R^**−**^, n = 8; No. 13 AT2R^**+**^, n = 5; No. 37 AT2R^**−**^, n = 7; No. 37 AT2R^**+**^, n = 5; No. 42 AT2R^**−**^, n = 7; No. 42 AT2R^**+**^, n = 5; No. 44 AT2R^**−**^, n = 8; No. 44 AT2R^**+**^, n = 5; No. 51 AT2R^**−**^, n = 5; No. 51 AT2R^**+**^, n = 5; No. 56 AT2R^**−**^, n = 7; No. 56 AT2R^**+**^, n = 5; No. 63 AT2R^**−**^, n = 6; No. 63 AT2R^**+**^, n = 5). Log-rank test. F, Flow cytometry analysis of long-term engraftment of non-CD34-expressing human AML in NSG mice (n = 7 cases of AML: 1, 4, 7, 10, 18, 22, 24, 25, 26, 29, 38, 40, 49, 52, 54, 55, 58, 62, 65, 69, 71. n = 8 mice). G, Flow cytometry analysis of AT2R^**−**^ AML cells from engrafted mice by non-CD34-expressing human AML (post-transplantation) (n = 7 cases of AML: 1, 4, 7, 10, 18, 22, 24, 25, 26, 29, 38, 40, 49, 52, 54, 55, 58, 62, 65, 69, 71. n = 8 mice). H, Representative plots showing AT2R expression in a high-CD34-expressing AML sample (No. 7). I, Quantification of in vitro CFU assay of high-CD34-expressing human AML (n = 5 cases of AML: 7, 18, 38, 49, 54). Connected dots depict colony-forming units of CD34^**+**^AT2R^**+**^ or CD34^**+**^AT2R^**−**^ fractions from one sample. J, Survival curve of high-CD34-expressing human AML-transplanted mice (n = 5 cases of AML. Transplanted mice: No. 7 CD34^**+**^AT2R^**−**^, n = 8; No. 7 CD34^**+**^AT2R^**+**^, n = 5; No. 18 CD34^**+**^AT2R^**−**^, n = 5; No. 18 CD34^**+**^AT2R^**+**^, n = 5; No. 38 CD34^**+**^AT2R^**−**^, n = 6; No. 38 CD34^**+**^AT2R^**+**^, n = 5; No. 49 CD34^**+**^AT2R^**−**^, n = 6; No. 49 CD34^**+**^AT2R^**+**^, n = 5; No. 54 CD34^**+**^AT2R^**−**^, n = 6; No. 54 CD34^**+**^AT2R^**+**^, n = 5). Log-rank test. K, Flow cytometry analysis of long-term engraftment of high-CD34-expressing human AML in NSG mice (n = 5 cases of AML: 7, 18, 38, 49, 54. n = 8 mice). Unless noted, each dot represents an individual sample or mouse, horizontal bars and whiskers denote mean with IQR, and *P* values are annotated and calculated using student’s t test.

While CD34 marks LSC-enriched population, some AML cases show low or absent CD34 expression^16^. We studied 7 CD34-non-expressing AML samples, which showed similar heterogeneous expression of AT2R to CD34-expressing AML (Fig. S2A). We analyzed other LSC markers and found AT2R^−^ cells are also enriched in CD117^+^ and CD123^+^ populations in all 62 AML samples (Fig. 3A and B). AT2R^−^ cells are also smaller in size, with less granularity (Fig. 3C), supporting that they are enriched with LSCs. Similar to CD34-expressing AML cells, CD34-non-expressing AT2R^−^ cells showed higher clonogenicity (Fig. 3D) and specific ability to repopulate immunodeficient mice (AT2R^−^ cells, 56 engrafted out of 56 transplanted mice (100%); AT2R^+^ cells, 0 engrafted out of 56 transplanted mice (0%); Fig. 3E and F; Fig. S2B to D). AT2R^−^ cells gave rise to both AT2R^−^ and AT2R^+^ progeny in engrafted mice and show enrichment in the BM (Fig. 3G).

In AML samples that display high expression of CD34 (Fig. 3H; Fig. S2E), CD34^+^AT2R^+^ showed no clonogenicity and in vivo AML engraftment (Fig. 3J and K; Fig. S2F and G), suggesting AT2R is a potential marker to stratify and enrich LSCs in both CD34-expressing and -non-expressing AML cases.

### *AGTR2* is a potential tumor suppressor silenced by chromatin reorganization and epigenetic mechanisms

**Fig. 4:**
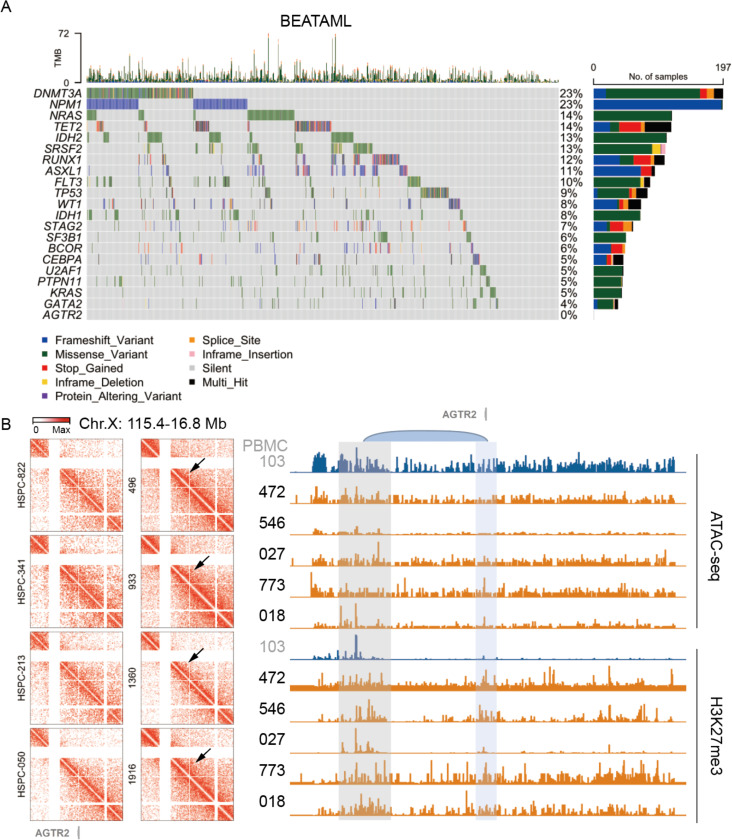
*AGTR2* is a potential tumor suppressor silenced by chromatin reorganization and epigenetic mechanisms A, Oncoprint plot showing the distribution and frequency of recurrent somatic mutations across 871 AML patient samples in the BEATAML cohort. Each column represents an individual patient, and each row represents a gene. Top 20 mutated genes and *AGTR2* are shown. Mutation types are color-coded as indicated. The top panel shows tumor mutational burden (TMB) per sample. The bar plot on the right depicts the percentage and number of cases harboring mutations in each gene. B, Left shows Hi-C matrix of human HSPC (left) or AML (right) surrounding of *AGTR2*. Right, from top to bottom, shows the genome browser tracks for ATAC-seq and H3K27me3 of healthy individual PBMC (No. 103) and AML. The gray arcs mark the loop anchors, which link the *AGTR2* to distal enhancers.

The ubiquitous downregulation of AT2R expression in AML suggests AT2R may function as a tumor suppressor. To investigate the mechanism of AT2R suppression, we first analyzed whole-exome sequencing data from the BEATAML and TGCA cohorts (Fig. 4A; Fig. S3A). Recurrent somatic mutations were observed in known AML drivers, including *DNMT3A*, *NPM1*, *NRAS*, and *TET2*. Notably, *AGTR2* did not harbor coding mutations in any samples, suggesting that it may be regulated by non-genetic mechanisms. Tumor suppressors are known to be suppressed by genetic and epigenetic mechanisms in cancer^17^. To examine the chromatin and epigenetic status of the *AGTR2* locus, we analyzed Hi-C, H3K27me3 CUT&Tag, and ATAC-seq profiles from HSPCs/PBMCs and primary AML patient samples (Fig. 4B, left)^18^. In HSPCs, Hi-C maps revealed a defined topologically associating domain (TAD) surrounding *AGTR2*, consistent with an intact chromatin architecture supportive of gene expression.

In contrast, AML samples show specific loops between *AGTR2* and a cluster of co-occurring silencers around 250 kb upstream, marked by increased deposition of H3K27me3 (Fig. 4B, right), indicating Polycomb-mediated transcriptional repression, and reduced chromatin accessibility based on ATAC-seq signal (Fig. 4B, right), suggesting that the locus becomes physically inaccessible. These changes were consistent across multiple AML patients and absent in healthy PBMCs, where *AGTR2* remains accessible and lacks repressive marks. Together, these data indicate that *AGTR2* may function as a tumor suppressor in AML, and is transcriptionally silenced not by mutation, but through chromatin reorganization and coordinated epigenetic repression involving both Polycomb-mediated histone modification and decreased chromatin accessibility.

### *Agtr2* is a functional tumor suppressor in mouse AML models

To further evaluate the role of AT2R as a tumor suppressor in AML, we generated two primary mouse AML models. Lin^−^ (B220^−^ CD19^−^ CD3^−^ CD8^−^ CD11b^−^ Gr-1^−^ TER119^−^ NK1.1^−^) cells from C57/B6 donor mice infected by the retroviral oncogene (MLL-AF9; MA9^19^ or AML1-ETO9a; AE9^20^)−internal ribosome entry site (IRES)−green fluorescent protein (GFP) were used to induce AML as previously described (Fig. S4A)^21,22^. Interestingly, we observed a gradual decrease in AT2R after retrovirus infection in primary transplant (Fig. S4B to D). We then generated three different shRNAs targeting *AGTR2* to functionally study the role of AT2R in AML. All shRNAs efficiently decreased the expression of *Agtr2* (Fig. S4E). The one with the strongest knockdown effects (sh-*Agtr2*-1) was carried on to the following experiments and transduced into AML cells isolated from the primary transplant. Transduced (*Agtr2*-KD) AML cells are then injected into secondary transplant mice. The recipient mice transplanted with *Agtr2*-KD AML cells show faster leukemia development and shorter survival in both MA9 (median survival of days, control 33.5 vs *Agtr2*-KD 31.5; 92.9% (BM), 72.6% (liver; LV), 84.4% (peripheral blood; PB), and 59.6% (spleen, SP) increase in ratios of leukemia cells in *Agtr2*-KD vs control) and AE9 AML (median survival of days, control 87.5 vs *Agtr2*-KD 66.5; 88.2% (BM), 321.1% (LV), 133.2% (PB), and 250.5% (SP) increase in ratios of leukemia cells in *Agtr2*-KD vs control) models.

We next generated a hematopoietic system-specific enforced expression of *Agtr2* in mice to study its role in both normal hematopoiesis and AML development^23,24^. The *Agtr2* transgenic (TG) mice were crossed with HSC-SCL-CreER^T^ and treated with tamoxifen (TMX) to specifically induce AT2R overexpression in hematopoietic cells (*Agtr2*^o/e^ mice) (Fig. S4F). *Agtr2*^o/e^ mice had comparable frequency and total numbers of HSPCs and downstream progenitor cells (Long-term hematopoietic stem cell, LT-HSC; short-term HSC, ST-HSC; multiple potential progenitors, MPP; Common Myeloid Progenitor, CMP; Granulocyte-Macrophage Progenitor, GMP; Common Lymphoid Progenitor, CLP) in the BM (Fig. S4G to H) and comparable cell cycle activity of HSCs (Fig. S4I and J) when compared with *Agtr2*^+/+^ mice. Functionally, *Agtr2*^o/e^ BM cells had comparable colony-forming ability (Fig. S4K) and engraftment ability with no biased lineage reconstitution (Fig. S4L and M) with *Agtr2*^+/+^ BM cells.

Then, *Agtr2*^o/e^ or littermate control mice were used to generate MA9 or AE9 AML (*Agtr2*^o/e^ AML or WT AML mice) to transplant into C57/B7 recipients (Fig. S4A). The recipient mice transplanted with *Agtr2*^o/e^ AML cells show reduced leukemia development and longer survival in both MA9 (median survival of days, WT 36 vs *Agtr2*^o/e^ 76.5; 65.1% (BM), 65.2% (LV), 63.9% (PB), and 60.2% (SP) reduction in ratios of leukemia cells in *Agtr2*^o/e^ vs WT) and AE9 AML (median survival of days, WT 81 vs *Agtr2*^o/e^ 146; 69.7% (BM), 64.7% (LV), 72.2% (PB), and 69.2% (SP) reduction in ratios of leukemia cells in *Agtr2*^o/e^ vs WT) models.

**Fig. 5:**
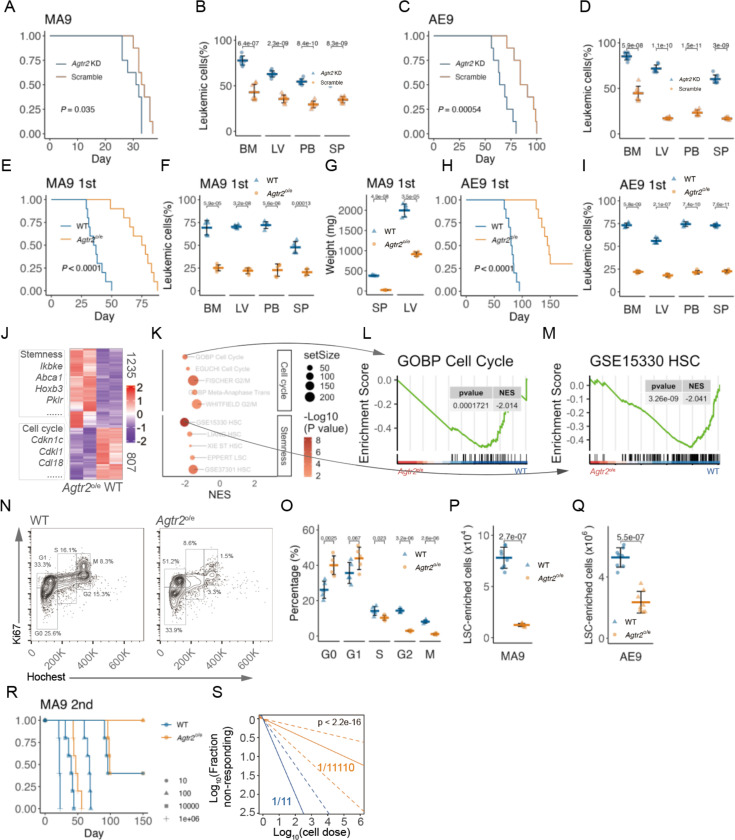
AT2R is a functional tumor suppressor in mouse AML models A, Survival curve of mice receiving *Agtr2* knockdown (KD) or control (Scramble) MLL-AF9 AML cells (n = 8 mice). B, Flow cytometry analysis of the percentages of AML cells in the BM, SP, LV, and PB of mice transplanted with *Agtr2* knockdown (KD) or control (Scramble) MLL-AF9 AML 4 weeks after transplantation (n = 8 mice). C, Survival curve of mice receiving *Agtr2* knockdown (KD) or control (Scramble) AML1-ETO9Aa AML cells (n = 8 mice). D, Flow cytometry analysis of the percentages of AML cells in the BM, SP, LV, and PB of mice transplanted with *Agtr2* knockdown (KD) or control (Scramble) AML1-ETO9Aa AML 7 weeks after transplantation (n = 8 mice). E, Survival curve of mice receiving MLL- AF9-infected WT and *Agtr2*^*o*/e^ HSPCs (n = 8 mice). F, Flow cytometry analysis of the percentages of AML cells in the BM, SP, LV, and PB of mice transplanted with MLL- AF9-infected WT and *Agtr2*^*o*/e^ HSPCs 6 weeks after transplantation (n = 8 mice). G, SP and LV weights of mice transplanted with MLL- AF9-infected WT and *Agtr2*^*o*/e^ HSPCs 6 weeks after transplantation (n = 8 mice). H, Survival curve of mice receiving AML1-ETO9Aa-infected WT and *Agtr2*^*o*/e^ HSPCs (n = 8 mice). I, Flow cytometry analysis of the percentages of AML cells in the BM, SP, LV, and PB of mice transplanted with AML1-ETO9Aa-infected WT and *Agtr2*^*o*/e^ HSPCs 10 weeks after transplantation (n = 8 mice). J, Heatmap of DEGs (1.5 FC, P < 0.05) from RNA-seq of WT and *Agtr2*^*o*/e^ MLL-AF9 AML cells (n = 2 groups). Genes of interest are highlighted. K to M, Dot plot of gene set enrichment analysis (GSEA) (K) and GSEA plot (L and M) illustrating the biological processes associated with enforced *Agtr2* expression. P values are annotated with a colored scheme. NES, normalized enrichment score. N and O, Representative plot (P) and quantification (Q) of flow cytometry analysis of the cell cycle progression of WT and *Agtr2*^*o*/e^ MLL-AF9 AML cells (n = 5 mice). P and Q, Absolute numbers of LSC-enriched population in the BM of WT and *Agtr2*^*o*/e^ MLL- AF9 (P) and AML1-ETO9Aa (Q) AML mice (n = 5 mice). R and S, Survival curve of mice receiving different doses of WT or *Agtr2*^*o*/e^ MLL-AF9 AML cells (n = 5 mice) and associated limiting dilution plot, where expected frequencies of LSCs are annotated (S). Unless noted, each dot represents an individual mouse, horizontal bars and whiskers denote mean and SD, and *P* values are annotated and calculated using student’s t test.

To determine the mechanisms by which AT2R regulates AML development, we analyzed genome-wide transcriptome changes in *Agtr2*^+/+^ versus *Agtr2*^o/e^ MLL-AF9 AML cells. This procedure yielded a gene expression signature consisting of 1235 upregulated and 807 downregulated genes (1.5-FC, *P* < 0.05). Gene signatures associated with AML stemness and cell cycle activity were downregulated in *Agtr2*^o/e^ AML (MSigDB, http://software.broadinstitute.org/gsea/msigdb/search.jsp ; Fig. 5J to M). We then evaluated AML cell cycle and found that AML cells in *Agtr2*^o/e^ mouse BM are less in the S (10.3±1.3% vs 14.1±2.5%), G2 (2.9±0.5% vs 14.3±1.2%) and M (1.1±0.5% vs 8.1±0.8%) phases but more in G0 (40.1±5.2% vs 26.1±4.8%) and G1 (43.9±6.3% vs 35.6±5.9%) phases, highlighting cell cycle arrest at G0/G1 phase (Fig. 5N and O). The LSC-enriched populations (L-GMP for MA9^19^ and LSK for AE9^20^) are reduced in *Agtr2*^o/e^ AML mice at a similar stage of AML development when compared with WT AML mice (56.3% and 36.6% reduction in MA9 and AE9, respectively; Fig. 5P and Q; Fig. S4N and O). Next, we relied on functional assays to evaluate the activity of LSCs. Our limiting dilution assays showed that leukemia-initiating cells are substantially reduced in *Agtr2*^o/e^ AML mice (Fig. 5R to U).

### Enforced AT2R expression inhibits fatty acid oxidation in mouse AML Cells

**Fig. 6:**
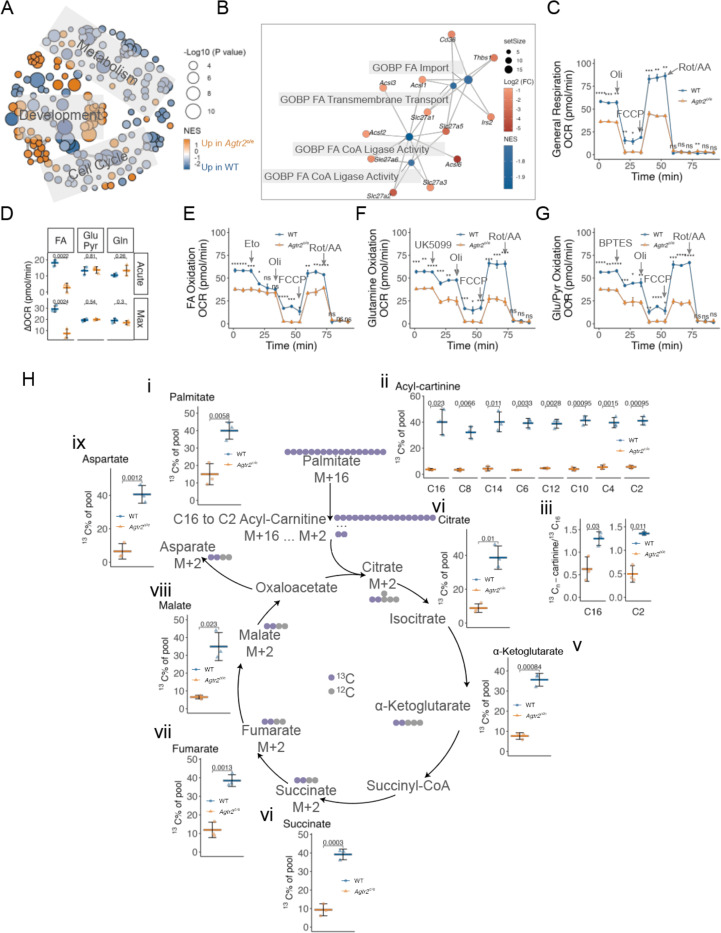
Enforced AT2R expression inhibits fatty acid oxidation in mouse AML Cells A, Enrichment map analysis of leading edge shows significantly enriched pathways, including reduced metabolism and cell cycle, in *Agtr2*^**o/e**^ compared with WT AML. The distance reflects the connectivity between each node. B, Cnet plot of enriched FA metabolism pathways showing reduced FA transportation and CoA ligation. Nodes represent genes and pathways, and edges represent the linkages between them. C to E, Oxygraph (C and E) and quantification (D) of basal and maximal respiration of WT and *Agtr2*^*o*/e^ MA9 AML cells (n = 3 mice). In brief, following injection into the high-resolution respirometer, cells demonstrate a basal rate of respiration. Specific metabolism was not inhibited (General respiration) or inhibited by Etomoxir (Eto, FA metabolism), UK5099 (Glutamine metabolism), or BPTES (Glycolysis). ATP synthesis is uncoupled by addition of oligomycin (Oli). Maximal respiration is stimulated with an injection of a chemical uncoupler (FCCP). All mitochondrial respiration is inhibited by addition of rotenone and antimycin A (Rot/AA). H, Levels of ^**13**^C-labeled Palmitate (i), Acyl-carnitine (ii), and enrichment of ^**13**^C atoms into TCA metabolites (iii to ix) in MA9 AML cells after 12 hours of incubation (n = 3 mice).

To further understand the biological functions induced by enforced *Agtr2* expression, we performed leading-edge network analysis using the gene sets mentioned above (MSigDB; Fig. 6A). The top 200 altered gene sets are associated with cell development, cell cycle, and metabolism. Gene sets associated with cell development showed both increased and reduced enrichment and, consistent with our prior findings (Fig. 5), gene sets associated with cell cycle predominantly showed reduced enrichment in *Agtr2*^o/e^ AML cells (Fig. 6A). Gene sets associated with metabolism are mostly reduced in *Agtr2*^o/e^ AML, in which the profoundly reduced are biological processes associated with fatty acid (FA) metabolism, including FA transportation (marked by downregulation of *Slc27* family genes) and FA-CoA ligation (marked by downregulation of *Acsl* family genes) (Fig. 6B). These are indispensable steps required for FA oxidation. We then assessed mitochondrial function using Seahorse assays and found *Agtr2*^o/e^ AML cells exhibited significantly diminished general respiration (Fig. 6C). To confirm the reduced FA utilization, we measured real-time mitochondrial respiration under substrate-specific conditions. *Agtr2*^o/e^ cells displayed significantly inhibited FA-specific OCR, which was reduced upon inhibition of carnitine palmitoyltransferase 1 (CPT1) by etomoxir (Fig. 6D and E), indicating enhanced CPT1-dependent mitochondrial FA import. In contrast, glutamine- and glucose/pyruvate-supported respiration were minimally changed in *Agtr2*^o/e^ cells (Fig. 6F and G), suggesting a selective metabolic inhibition of FAO. To specifically dissect which step of FA oxidation was affected, we performed stable isotope tracing using ^13^C-palmitate (Fig. 6H). Consistent with our leading-edge analysis, *Agtr2*^o/e^ AML cells demonstrated markedly reduced intracellular import of FA (Fig. 6H, i) as well as FA-CoA ligation (shown by incorporation of ^13^C into long-chain acyl-carnitines (Fig. 6H, ii and iii)). This resulted in ubiquitously reduced downstream metabolites, including α-ketoglutarate, succinate, fumarate, malate, and aspartate in *Agtr2*^o/e^ AML cells (Fig. 6H, iv–ix). Together, these data reveal a broad suppression of mitochondrial FA oxidation in *Agtr2*^o/e^ AML.

### Re-activation of *Srebf1* rescued FA oxidation inhibition induced by enforced *Agtr2* expression

**Fig. 7:**
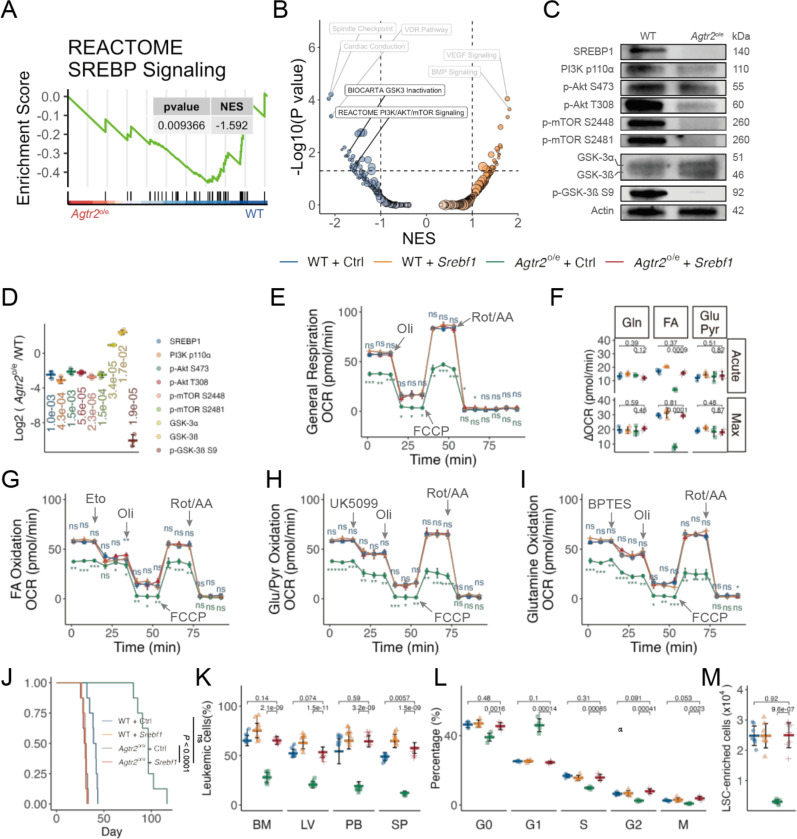
Re-activation of *Srebf1* rescuced FA oxidation inhibition induced by enforced *AGTR2* expression A, GSEA plot shows significant downregulation of SREBP1 pathway gene signatures upon enforced *Agtr2* expression. NES, net enrichment score. B, Volcano plot showing altered signaling pathways gene signatures upon enforced *Agtr2* expression. C and D, Representative plot (C) and quantification of western blot analysis of the indicated proteins in WT and *Agtr2*^*o*/e^ MLL-AF9 AML cells. β-Actin was used as the loading control. E to I, Oxygraphy (E and I) and quantification (D) of basal and maximal respiration of WT and *Agtr2*^*o*/e^ MA9 AML cells with or without re-introduction of *Srebf1* (n = 3 mice). In brief, following injection into the high-resolution respirometer, cells demonstrate a basal rate of respiration. Specific metabolism was not inhibited (General respiration) or inhibited by Etomoxir (Eto, FA metabolism), UK5099 (Glutamine metabolism), or BPTES (Glycolysis). ATP synthesis is uncoupled by addition of oligomycin (Oli). Maximal respiration is stimulated with an injection of a chemical uncoupler (FCCP). All mitochondrial respiration is inhibited by addition of rotenone and antimycin A (Rot/AA). J, Survival curve of mice receiving WT and *Agtr2*^*o*/e^ MA9 AML cells with or without re-introduction of *Srebf1* (n = 8 mice). K, Flow cytometry analysis of the percentages of AML cells in the BM, SP, LV, and PB of mice transplanted with WT and *Agtr2*^*o*/e^ MA9 AML cells with or without re-introduction of *Srebf1* 6 weeks after transplantation (n = 8 mice). L, Quantification of flow cytometry analysis of the cell cycle progression of WT and *Agtr2*^*o*/e^ MA9 AML cells with or without re-introduction of *Srebf1* (n = 5 mice). M, Absolute numbers of LSC-enriched population in the BM of mice transplanted with WT and *Agtr2*^*o*/e^ MA9 AML cells with or without re-introduction of *Srebf1* (n = 5 mice). Unless noted, each dot represents an individual mouse, horizontal bars and whiskers denote mean and SD, and *P* values are annotated and calculated using student’s t test.

We then dissected possible signaling pathways that are responsible for the observed AML phenotypes and altered FA metabolism induced by enforced *Agtr2* expression in our gene set enrichment analysis (GSEA) results. In line with the reduced FA metabolism, we observed downregulated SREBP signaling in *Agtr2*^o/e^ AML cells (Fig. 7A). We did not observe a significant change in SREBP transcription levels (Fig. S5A). However, we found reduced protein expression of SREBP1, which is known to regulate lipogenic gene expression^25^. To understand the mechanism of reduced SREBP1 expression, we explored other signalings and found reduced PI3K/Akt/mTOR signaling and increased GSK3 signaling (Fig. 7B)^25,26^. PI3K/Akt/mTOR is responsible for SREBP1 activation. Indeed, we observed reduced PI3K p110ɑ, reduced Akt phosphorylation at S473 and T308, and reduced mTOR phosphorylation at S2448 and S2481 (Fig. 7C and D). GSK3 is responsible for SREBP1 deactivation and its activity is inhibited by phosphorylation. We observed increased total GSK3 level and reduced GSK3ß phosphorylation (Fig. 7C and D). To functionally confirm the role of SREBP1 in the phenotypes observed in *Agtr2*^o/e^ AML, we performed rescue assays where *Srebf1* was reintroduced into *Agtr2*^o/e^ MLL-AF9 mouse AML cells before transplant into recipient mice (Fig. S5B). Phenotypes associated with enforced *Agtr2* expression, including altered cell metabolism (Fig. 7E to I), mouse survival (Fig. 7J), leukemic burden (Fig. 7K), AML cell cycle (Fig. 7L), and AML stemness (Fig. 7M) were rescued by re-introduction of *Srebf1*, indicating AT2R activation suppressed SREBP1 activation to limit FA utilization in AML cells.

### Enforced Agtr2 expression disrupts enhancer loops of genes associated with leukemia stemness

Recent studies have shown that AML hijacks histone enhancers to enforce the expression of genes associated with leukemia stemness^18^. In addition, altered metabolism is often associated with chromatin rearrangement^27^ , and our data shows chromatin organization and epigenetic mechanisms are crucial for *Agtr2* expression (Fig. 4), highlighting the importance of three-dimensional (3D) genomic structure changes in AML and necessitating a deeper understanding of the underlying mechanisms.

**Fig. 8:**
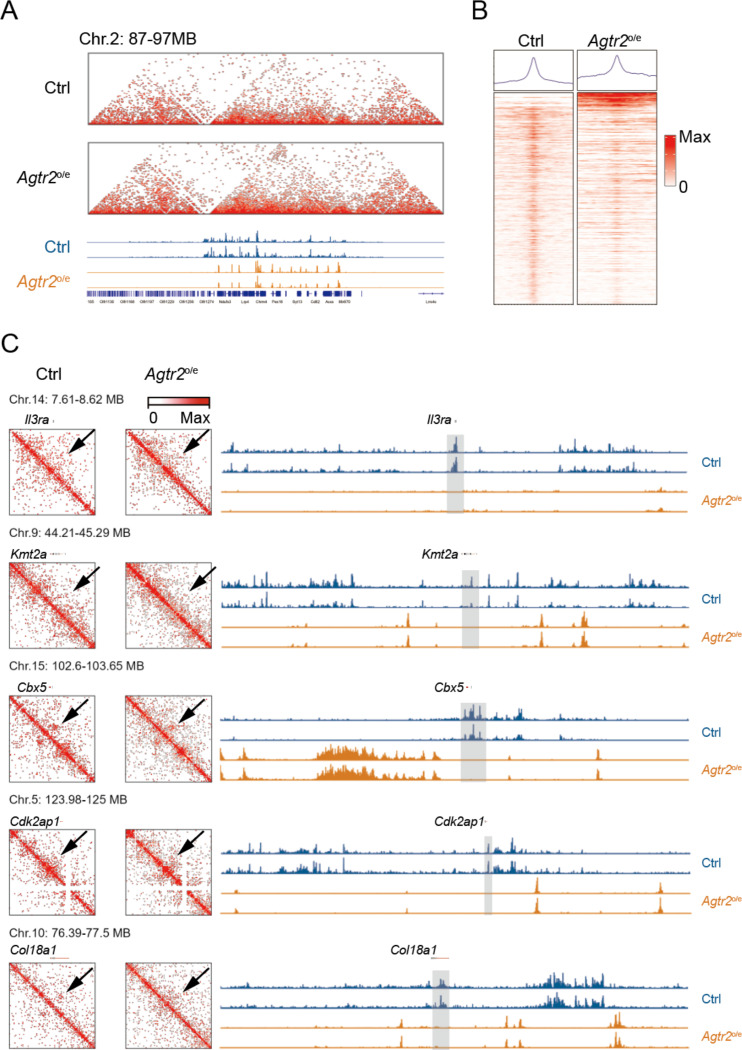
SREBP1 signaling regulates three-dimensional genomic structure of genes associated with LSCs A, Snapshot of an example region shows Hi-C and CUT&TAG for H3K27ac in the same region of a WT and *Agtr2*^*o*/e^ MA9 AML. Values for the y axis for the data were normalized to sequencing depths. B, Heatmaps of CUT&TAG profiling for H3K27ac. C, Left, Hi- C matrix surrounding of the indicated genes. Right, from top to bottom, shows the genome browser tracks for H3K27ac of WT and *Agtr2*^*o*/e^.

We conducted in situ Hi-C and CUT&Tag assays of histone 3 lysine 27 acetylation (H3K27ac) using AML cells from WT and *Agtr2*^o/e^ MA9 AML mice. Fig. 8A illustrates a region containing all the epigenetic data in the WT and *Agtr2*^o/e^ AML cells. Our data revealed altered histone enhancer (H3K27ac) expression spectrum, with reduced overall binding sites in *Agtr2*^o/e^ AML cells (Fig. 8B). In WT AML cells, genes associated with LSC stemness, including *Il3ra*, *Kmt2a*, *Cbx5*, *Cdk2ap1*, and *Col18a1*, formed a loop with co-occurring enhancers and showed marks of H3K27ac (Fig. 8C)^28–30^. However, such loops and enhancer marks were absent in *Agtr2*^o/e^ AML cells. These findings demonstrate that *Agtr2* overexpression disrupts enhancer loops in AML and impairs leukemia stemness.

### A phase II-like preclinical trial of buloxibutid in AML PDX

**Fig. 9:**
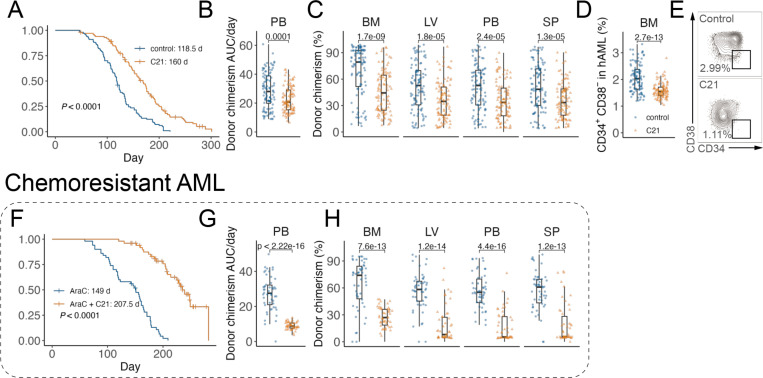
A phase II-like preclinical trial of buloxibutid in AML PDX A, Survival analysis of vehicle control or C21-treated AML PDX models (n = 20 cases of AML: 3, 6, 9, 18, 25, 26, 28, 37, 38, 40, 42, 46, 51, 53, 55, 58, 60, 69, 70, 71. n = 5 mice per sample). Log-rank test. B and C, Peripheral blood (PB) donor chimerism area under the curve (AUC) per day (B) and end point hematopoietic organ donor chimerism (C) (n = 20 cases of AML: 3, 6, 9, 18, 25, 26, 28, 37, 38, 40, 42, 46, 51, 53, 55, 58, 60, 69, 70, 71. n = 5 mice per sample). D and E, Flow cytometry analysis of AML surface marker expression of CD34 and CD38 (D) and representative plot (E). A, Survival analysis of AraC control or AraC+C21-treated chemo-resistant AML PDX models (n = 210 cases of AML 10, 24, 38, 39, 47, 52, 55, 63, 71, 72. n = 5 mice per sample). Log-rank test. B and C, Peripheral blood (PB) donor chimerism area under the curve (AUC) per day (B) and end point hematopoietic organ donor chimerism (C) (n = 210 cases of AML: 10, 24, 38, 39, 47, 52, 55, 63, 71, 72. n = 5 mice per sample). Unless noted, each dot represents an individual mouse, horizontal bars and whiskers denote mean with IQR, and *P* values are annotated and calculated using student’s t test.

Finally, to test the preclinical efficacy of C21 in AML, 20 characterized individual samples from patients with AML were each transplanted into 10 NSG recipients (n = 200 PDXs in total). Once the AML burden was detected, PDXs were randomized and treated with C21 or vehicle control (PBS) until disease onset or a survival benefit of at least 30 days was reached. Median survival was significantly prolonged in C21 compared to PBS-treated PDXs (160 vs 118.5 days after start of treatment, P < 0.0001; Fig. 9A; Fig. S5A). AML burden measured as PB donor chimerism per day was significantly lower in C21 compared to PBS-treated recipients (Fig. 3B; Fig. S5B). Moreover, end-point BM, LV, PB, and SP donor chimerism were significantly reduced in recipients treated with C21 when compared to vehicle control (Fig. 9C; Fig. S5C). We next assessed AML surface marker expression associated with LSCs^31^. C21 significantly diminished the CD34^+^CD38^−^ LSC-enriched AML cell population (Fig. 9D and E).

We next aimed to evaluate C21’s efficacy against chemo-resistant AML. We selected 10 individual samples obtained from patients with standard induction chemotherapy or from relapsed AML patients. In in vitro culture assay with chemotherapy alone (AraC) or combination treatment (AraC + C21), AML samples show minimal response to AraC when compared with control (PBS-treated) AML, while combo treatment resulted in reduced cell growth (Fig. 9F). We then transplanted each AML sample into 10 NSG recipients (n = 100 PDXs in total). In vivo, AraC + C21 conferred a significantly greater survival advantage (207.5 vs 149 days, P < 0.0001; Fig. 9G; Fig. S5D) compared with AraC alone. PB donor chimerism per day was significantly decreased in recipients treated with the combination compared to AraC alone (Fig. 9H; Fig. S5E). Similarly, endpoint BM, LV, PB, and SP donor chimerism were consistently lower in AraC + C21-treated animals compared to AraC alone (Fig. 9I; Fig. S5F). Together, these data demonstrate that buloxibutid not only improves survival and reduces AML burden in diverse patient-derived xenograft models but also enhances chemotherapy efficacy in chemo-resistant AML.

## Discussion

Recent advances in LLMs are beginning to reshape how therapeutic targets are nominated and prioritized in cancer research. In this study, we integrated a tool-augmented LLM agent into the discovery pipeline to systematically evaluate potential AML targets. Rather than relying solely on survival stratification – which is confounded by heterogeneity in therapy and comorbidities- we trained the agent to leverage ELN 2022 risk categories and therapeutic response as clinically grounded endpoints. The LLM framework enabled us to combine primary signals from our AML cohort with curated external knowledge bases, performing a multi-step evaluation that spanned druggability, survival, and dependency cross-validation, literature synthesis, and human genetics safety filtering. This yielded a transparent, ranked list of candidate targets, complete with structured dossiers that contextualized oncogenic roles, therapeutic relevance, novelty, and toxicity risks. Such an approach demonstrates the feasibility of harnessing generative AI not only for hypothesis generation but also for creating traceable, evidence-based target nomination in hematologic malignancies.

Importantly, the agent-based pipeline converged on several candidates that aligned with our biological findings, including AT2R, as well as other lineage-selective or understudied targets (e.g., NTRK1, LAG3, MPL, TERT). The concordance between computational prioritization and experimental validation underscores the potential of LLM-guided frameworks to accelerate discovery and reduce investigator bias. Furthermore, the explicit integration of safety metrics – such as gnomAD constraint, GTEx expression, and eQTL breadth – provides a safeguard against nominating broadly expressed or high-toxicity targets, a limitation of many conventional bioinformatics screens. By incorporating such multi-layered filters, the LLM agent helped to highlight novel tumor suppressive pathways that may otherwise be overlooked due to their unconventional biology. More broadly, this work illustrates how AI-driven approaches can serve as powerful adjuncts to experimental investigation, bridging patient-derived data, knowledge bases, and mechanistic validation to deliver clinically relevant insights.

In this study, we reveal a previously unappreciated tumor suppressor pathway in AML centered on the AT2R and demonstrate a novel therapeutic approach to exploit this pathway. Our findings show that loss of AT2R expression marks a functionally distinct subpopulation of AML cells with leukemia stem cell properties and chemotherapy resistance. AT2R^–^ cells across different AML patient samples were highly enriched for LSC activity: they were capable of long-term engraftment in mice and survived cytotoxic chemotherapy, whereas AT2R^+^ cells were largely non-engrafting and chemosensitive. These data identify AT2R negativity as a reliable phenotypic marker of high-risk LSCs in both conventional (CD34^+^) and atypical (CD34^–^) AML contexts. Consistent with this, we found that AML patients with higher AT2R expression tend to have better responses to therapy and outcomes, suggesting that AT2R expression (or lack thereof) could have prognostic significance. From a clinical perspective, assessing AT2R levels in AML might help stratify patients by the aggressiveness of their LSC compartment – patients with AT2R-low/negative LSCs may be at greater risk of relapse and could be candidates for intensified or alternative therapies.

Mechanistically, our work establishes AT2R as a bona fide tumor suppressor in AML that is silenced via epigenetic means. Unlike many genetic drivers of leukemia, we found no recurrent mutations in *AGTR2* in large AML cohorts, implying no positive selection for coding alterations. Instead, AML cells selectively repress AT2R through chromatin reorganization: the formation of a repressive chromatin loop engaging AT2R with upstream H3K27me3-marked elements effectively shuts down gene transcription. This mode of inactivation is reminiscent of other tumor suppressors that are silenced by polycomb complexes or DNA hypermethylation in AML, e.g., CDKN2A/B or WT1^32,33^. Our data add a new dimension by showing a 3D genome wiring change as a driver of *AGTR2* silencing – a strategy that leukemia cells appear to use to lock away a gene that is deleterious to their maintenance. It will be interesting in future studies to determine if this structural silencing of AT2R is reversible. Agents such as EZH2 inhibitors or DNA hypomethylating agents could potentially restore AT2R expression in AT2R^–^ LSCs, which might sensitize them to AT2R-targeted treatments. The epigenetic plasticity of the *AGTR2* locus also underscores how dynamic the leukemia genome is in sculpting its regulatory landscape to favor malignancy.

Through genetic models, we demonstrated that AT2R has potent anti-leukemic activity. AT2R loss-of-function accelerated leukemia progression and increased leukemic burden in mice, whereas AT2R gain-of-function drastically delayed AML development, reduced LSC frequency, and prolonged survival. These complementary approaches provide causative evidence that AT2R signaling constrains AML, functioning as a check on leukemia initiation and expansion. The fact that enforced AT2R expression had minimal impact on normal HSCs but a strong suppressive effect on leukemic cells suggests a possible therapeutic window: leukemia cells may be uniquely dependent on keeping AT2R silent, whereas normal hematopoiesis can tolerate AT2R activation. This selective pressure is likely why AML cells evolve to epigenetically silence *AGTR2*. Our results parallel observations in other cancers, e.g., solid tumors like prostate and bladder cancer, where AT2R activation triggers anti-proliferative effects^34^. However, to our knowledge, this is the first demonstration of AT2R’s role in a stem cell–driven malignancy and the first time AT2R has been shown to regulate cancer metabolism and epigenetic state.

One of the key discoveries here is that AT2R reactivation drives a comprehensive metabolic shutdown of fatty acid oxidation (FAO) in leukemia cells. LSCs and therapy-resistant AML blasts have been shown to rely on oxidative phosphorylation and FAO for survival^35–39^. By suppressing FAO, AT2R activation effectively starves these cells of an important energy source and building blocks for macromolecules. Indeed, our AT2R-overexpressing AML cells resembled cells treated with metabolic inhibitors: they had reduced respiratory capacity and lower levels of TCA cycle intermediates, indicating an energy deficit. This metabolic deficit likely underlies the observed cell-cycle arrest and loss of self-renewal – cells that cannot efficiently utilize fatty acids are less able to sustain the energetically demanding processes of proliferation and self-renewal^40^. Intriguingly, the way AT2R enforces this metabolic effect is through downregulation of SREBP1, a master regulator of lipid metabolism. Our data suggest that SREBP1 activity might be required for maintaining the cellular machinery or substrate availability for FAO via regulation of enzymes that channel fatty acids into mitochondria or by providing lipids that modulate mitochondrial function. By inhibiting PI3K/Akt/mTOR and activating GSK3, AT2R signaling tips the balance to an inactive SREBP1 state, thereby disturbing AML cells’ lipid metabolic network. This convergence on SREBP1 is notable because it provides a single molecular handle that ties together multiple phenotypic outcomes: metabolism, cell cycle, and chromatin regulation.

Our integrative analysis uncovered that SREBP1 activity – modulated by AT2R – influences the 3D genomic architecture of leukemia cells. Loss of SREBP1 led to the collapse of enhancer–promoter loops at critical stemness genes, whereas restoring SREBP1 reinstated those loops. One interpretation is that the metabolic state of the cell can dictate epigenetic configurations – when lipid metabolism is constrained, cells fail to sustain certain chromatin states^41^. Additionally, transcription factors or co-activators regulated by SREBP1 might be involved in maintaining those LSC gene enhancers^25,42^. It is also possible that SREBP1 itself, or a downstream lipid product, has a more direct role in chromatin organization. For example, nuclear SREBP1 might interact with chromatin remodelers, or lipid modifications of nuclear proteins could be required for chromatin looping. While the precise mechanisms warrant further investigation, our data clearly tie metabolic reprogramming to epigenetic reprogramming in AML. This aligns with a growing body of work showing that altering cancer metabolism can have epigenetic consequences, and vice versa, reinforcing the concept of “metabolic-epigenetic vulnerabilities” in cancer cells^43^. In the case of AT2R, leveraging this vulnerability means we might simultaneously disrupt LSC metabolism and the gene expression programs sustaining LSC identity.

From a translational standpoint, our preclinical therapy experiments with the AT2R agonist C21 are highly encouraging. Buloxibutid (C21) is an orally available, selective AT2R agonist that has shown safety and efficacy in models of fibrosis and is currently in clinical trials for idiopathic pulmonary fibrosis^7^. We demonstrated that C21 can be repurposed to target AML, producing meaningful therapeutic benefits in vivo. In a panel of heterogeneous patient-derived AML xenografts, AT2R activation via C21 significantly extended survival and reduced leukemia burdens, indicating on-target anti-leukemia activity across subtypes. Importantly, C21 also reduced the frequency of LSCs (CD34^+^CD38^−^ cells) in these models, which is a key requirement for preventing relapse. Perhaps the most striking result was the synergy between C21 and chemotherapy in refractory AML models. C21 converted chemo-resistant disease into chemo-responsive disease, enabling long-term remissions where chemotherapy alone had failed. This provides a proof-of-concept that AT2R agonists could be used as adjuncts to standard AML treatment to eliminate residual LSCs. Conceptually, this is analogous to the recent success of venetoclax (BCL-2 inhibitor) in combination with azacitidine for AML, which works by targeting the metabolic dependencies of LSCs^44^. AT2R agonism represents a different approach to hit LSC metabolism and could potentially be combined with existing regimens, including venetoclax-based therapy, for a multi-pronged attack on LSC energetics and survival pathways.

A critical question moving forward is how exactly AT2R agonists exert their effects in the complex human AML context. One consideration is that in our experiments, the LSCs themselves had low AT2R expression. If LSCs lack the target, drug efficacy is potentially hindered. Possible explanations include that: 1) a small subset of LSCs or progenitors do express AT2R at low levels and these are directly affected by C21, setting off a cascade that compromises the whole LSC pool; 2) AT2R agonism on more differentiated leukemia cells or other cells in the bone marrow microenvironment might create a less supportive niche for LSCs – for instance, by altering cytokine secretion or niche retention signals. We noted AT2R^+^ cells had reduced homing, which could translate to forcing LSCs out of their protective niches; 3) As our data showed, AT2R^–^ LSCs can give rise to AT2R^+^ progeny. By continuously activating AT2R, we may be eliminating the LSCs indirectly over time as they cycle through an AT2R-expressing state during differentiation. Determining which of these mechanisms (direct vs. indirect) predominates in AT2R agonist therapy will be important for optimizing its use.

We recognize that our study has several limitations and open questions. One limitation is that while we demonstrated the efficacy of AT2R activation in mice, the pharmacodynamics in humans might differ. AT2R is expressed in normal tissues, e.g., vasculature, kidney, and brain. Systemic AT2R agonism could have side effects such as hypotension, as high-dose C21 was reported to lower blood pressure in the TRAP prostate cancer model rats^45^. Encouragingly, clinical data from trials of C21 in fibrosis patients suggest it is well tolerated^7^, but AML patients might be more fragile, especially during chemotherapy. Thus, dosing and schedule will need careful refinement. Another question is how to best integrate AT2R-targeted therapy with existing treatments. Our data support combining AT2R agonists with chemotherapy. It would be logical to test combinations with venetoclax/azacitidine as well, since both target LSC metabolism, albeit via distinct pathways. It’s conceivable that AT2R agonism could prime LSCs for apoptosis by metabolic stress, enhancing the effect of venetoclax, which targets BCL-2-dependent OxPhos in LSCs^44^. There may also be a role for AT2R agonists in post-remission maintenance. Given their apparent ability to suppress residual LSCs without major toxicity, they could be used to maintain remission in patients not proceeding to transplant. Our “phase II-like” trial in mice effectively modeled such a scenario, showing prolonged leukemia-free intervals with continuous C21 treatment.

In terms of future directions, our findings open up several avenues. Investigating the precise signaling cascade from AT2R (a GPCR) to the PI3K/Akt/GSK3/SREBP1 axis in AML cells would deepen our understanding of LSC biology. AT2R is known in other contexts to signal through pathways involving protein phosphatases (like SHP-1), NO/cGMP, MAPK, and Akt^46–49^. It would be interesting to see if those intermediates are at play in AML. Additionally, exploring biomarkers of response to AT2R agonists will be important – for example, baseline AT2R levels or a transcriptional signature of AT2R pathway activation could identify patients most likely to benefit. From a broader perspective, our work highlights how hijacking developmental or homeostatic pathways (like the RAS-angiotensin system) can yield new cancer therapies. AT2R was not an obvious cancer target, yet reactivating it strikes at a fundamental vulnerability of leukemia cells – their need to preserve a stem-like state and metabolic flexibility. It is tempting to speculate that there may be other “dormant” tumor suppressive GPCR or signaling pathways in cancer cells that, if reawakened, could tip the balance towards differentiation or death of the malignant cells.

In conclusion, our study positions AT2R as a critical regulator of leukemia stemness and chemoresistance in AML. We provide a comprehensive analysis from patient samples to mechanistic mouse models and therapeutic trials, all converging on the insight that reactivating AT2R signaling in AML can disable LSCs and enhance treatment efficacy. These results lay the groundwork for clinical translation – for instance, a trial of an AT2R agonist like C21 in combination with chemotherapy in relapsed/refractory AML could be envisioned. If successful, such an approach could address one of the longest-standing challenges in AML therapy: the eradication of leukemia stem cells to achieve lasting remissions and cures. By targeting the “absence of AT2R” as a defining feature of chemo-resistant LSCs, we propose a novel angle of attack against AML, potentially improving outcomes for patients who currently face dismal prognoses due to relapse.

## Methods

### LLM agent

We developed a tool-augmented LLM agent to nominate and prioritize therapeutic targets in acute myeloid leukemia (AML). RNA-sequencing and clinical annotation data (GSE216738) from the AML cohort served as inputs, with ELN 2022 risk categories and therapeutic response used as clinically grounded endpoints in place of direct survival stratification. Differential expression analysis identified genes enriched in favorable versus adverse groups, and only protein-coding, cell-surface targets were retained to maximize druggability. For each candidate, the agent assembled a structured “target dossier” by integrating external resources: pharmacologic evidence (DGIdb, ChEMBL), interaction networks (STRING), CRISPR essentiality (DepMap), and survival cross-validation via elastic-net Cox modeling in the BEATAML dataset, further contextualized through pathway enrichment (Hallmark, Reactome). Literature mining was performed with the LLM to generate structured summaries of oncogenic role, therapeutic context, novelty, and key PubMed references. A genetics-based safety triage was then applied, incorporating gnomAD constraint metrics (pLI, LOEUF), GTEx transcript abundance across critical tissues, and cis-eQTL breadth, with penalties assigned to broadly expressed or dosage-sensitive genes. Finally, all features were aggregated into a composite priority score with safety penalties, producing a ranked target list and per-gene dossiers with transparent, traceable evidence. An implementation of our source code for researchers to extend on our work is available from GitHub (https://github.com/1boChen/gene-nlp ). Instructions for using the repository are detailed in the respective ReadMe file.

### Primary AML and healthy donor samples

A protocol approved by the University of Missouri (IRB #2018932) was used to obtain fresh human peripheral blood samples and primary human AML samples at the Ellis Fischel Cancer Center. Written informed consent was obtained from all patients to use their blood samples for research purposes. Diagnosis of AML was confirmed by a pathologist utilizing BM aspiration and biopsy and Cytochemical and Immunohistochemical tests. Cells were processed by density gradient centrifugation, viably frozen and freshly thawed for each experiment.

### Human AML xenograft

Adult NSG mice (6–8 weeks old) were sublethally irradiated with 250 cGy total body irradiation before transplantation. Bulk or sorted human AML cells were resuspended in 200ml PBS containing 2% FBS at a final concentration of 5x10^5^ viable cells per mouse for retro-orbital injection.

### Mice

C57B/6 mice were purchased from Charles River, Inc. *Agtr2* TG mice were gifts from Dr. Tadashi Yoshida. The offspring were then crossed with Scl-CreER^T^ mice (stock #037466)^24^, to obtain *Agtr2* TG; Scl-CreER^T^ mice. Mice were then induced with tamoxifen and then referred to as *Agtr2*^o/e.^ Reagents will be made available through a material transfer agreement.

### Leukemia characterization

After transplantation, survival were monitored and the numbers and infiltration of leukemia cells in PB, BM, SP, and LV were analyzed. Different populations of leukemia cells were characterized using flow cytometry. Moribund leukemic mice were euthanized, and the time was recorded as the time of death.

### Cell culture

293T cells were cultured in Dulbecco’s Modified Eagle Medium (DMEM) supplemented with 10% FBS at 37 °C in 5% CO2. Primary mouse AML cells were cultured in serum-free StemSpan (STEMCELL Technologies) supplemented with 50 ng/ml SCF, 10 ng/ml IL-3, 10 ng/ml IL-6 (STEMCELL Technologies). Human monocytic AML cells THP-1 (ATCC, TIB-202), macrophage B myelomonocytic leukemia MV4–11 (ATCC, CRL-9591), and histiocytic lymphoma U937 (ATCC, CRL-1593.2)) were cultured in Roswell Park Memorial Institute (RPMI) 1640 supplemented with 10% FBS at 37 °C in 5% CO_2_ and normal O_2_. All cell lines were routinely tested using a mycoplasma contamination kit (R&D Systems).

### AML transplantation

For primary transplantation, 2000 sorted infected GFP^+^ Lin^−^ cells along with 2 × 10^5^ BM cells were transplanted into lethally irradiated (1,000 cGy) C57BL/6 mice (6–8 weeks old) by retro-orbital injection. The engraftment was assessed every 2 weeks post-transplantation. For secondary transplantation, we used FACS to isolate GFP^+^ BM cells from primary recipient mice and transplanted 2,000 cells (MLL-AF9 model) together with 2 × 10^5^ normal BM cells into lethally irradiated recipients and engraftment assessed at 3 weeks post-transplantation.

### Colony-forming unit assay

Mouse AML cells were diluted to the indicated concentration in IMDM with 2% FBS and were then seeded into methylcellulose medium M3534 or M3434 or H4436 (STEMCELL Technologies) for myeloid colony formation analysis according to the manufacturer’s protocol.

### RNA-seq analysis

RNA obtained from sorted BM AML cells of two *Agtr2*^+/+^ and two *Agtr2*^o/e^ AML cells transplanted mice, was purified with QIAGEN miRNeasy Mini Kit. The sample concentration was determined by Qubit fluorometer (Invitrogen) using the Qubit HS RNA assay kit, and RNA integrity was assessed using the Fragment Analyzer automated electrophoresis system. Next, poly-A-containing mRNA was purified from total RNA (1mg), RNA was fragmented, double-stranded cDNA was generated from fragmented RNA, and the index-containing adapters were ligated to the ends. Libraries were constructed following the manufacturer’s protocol with reagents supplied in Illumina’s TruSeq mRNA stranded sample preparation kit. The amplified cDNA constructs were purified by the addition of Axyprep Mag PCR Clean-up beads. The final construct of each purified library was evaluated using the Fragment Analyzer automated electrophoresis system and quantified with the Qubit fluorometer using the Qubit HS dsDNA assay kit. Samples were pooled and sequenced on Illumina Novaseq X according to the manufacturer's recommendations to achieve 20 million paired reads per sample. The differentially expression genes were identified using standard DESeq2 workflow^50^.

### GSEA and leading-edge analysis

GSEA and leading-edge analysis were performed using clusterprofiler and R^51^. Gene sets were obtained from the MSigDB (http://software.broadinstitute.org/gsea/msigdb/search.jsp ).

ssGSEA is an extension of GSEA that calculates separate enrichment scores for each pairing of a sample and gene set. Each ssGSEA enrichment score represents the degree to which the genes in a particular gene set are coordinately upregulated or downregulated within a sample.

### In situ Hi-C

BM AML cells of two *Agtr1a*^+/+^ and two *Agtr1a*^−/−^ AML cells transplanted mice were washed twice with PBS, and 5–10 x 10^6^ cells were fixed in 4% paraformaldehyde/1x PBS for 15 min, quenched by the addition 0.125 M glycine final concentration for 5 min at room temperature. Thereafter, in situ Hi-C was performed using the Arima Hi-C kit (#A410030) according to the manufacturers’ recommendations. DNA input quantities ranged from approximately 0.9 – 3µg per replicate. Both proximity ligated DNA Hi-C samples passed QC1 checkpoint (high quality Arima-QC1 values are expected to be >15%). Next, output proximity ligated samples where mechanically fragmented by Covaris Ultrasonicator ME220, size selected to 200–600 bp (average fragment size of 400 bp) using AMPure XP Beads and monitored for correct size range by Agilent TapeStation 4200 analysis. After final biotin enrichment, processed Hi-C samples were converted to sequencing libraries with Arima library prep module (#A510008 and #A303011) according to the manufacturers’ guidelines with 7 cycles for the final PCR amplification step with Arima QC2 > 0.2% and sequenced with Illumina NovaSeq 6000 using 50 cycles paired- end mode.

### Hi-C data analysis

Paired-end reads were first trimmed by Trim_Galore! (v.0.6.0) to remove adapters and low-quality bases with parameter “--paired”. Trimmed reads were then mapped to mouse genome GRCm39 by BWA MEM (v.0.7.17-r1198) with parameter “-SP5M”, and deduplicated with “pairtools dedup” (v.0.3.0). Hi-C pairs were generated with “pairtools parse” by removing unmapped–multimapped read pairs and rescuing single ligations in chimeric reads. Reads mapped to the same MboI restriction fragment are not informative to chromatin interactions and therefore were removed for downstream analysis. Hi-C matrices and cooler files were generated with Cooler (v.0.8.6.post0)^52^. The iterative correction and eigenvector decomposition (ICE) method was used for Hi-C normalization with the “cooler balance” option. HiCool were used for visualization of Hi-C matrices^53^. Hi-C compartments were identified at 40-kb resolution using a “sliding window” strategy as previously described^54^. First, the “exp” (expected) matrix was obtained by averaging Hi-C contacts at the same distance. Then the “obs/exp” (observed/expected) matrix was calculated by summing the observed Hi-C contacts within a window of 400 kb centered at each bin divided by the sum of expected Hi-C contacts in the same window. A step size of 40 kb was used to calculate the obs/ exp value for all elements in the matrix. The obs/exp matrix was then converted to a Pearson’s correlation matrix. The principal components were derived by calculating the covariance matrix of the Pearson’s correlation matrix followed by eigenvector decomposition with the ‘eigen’ function in R. PC1 was used to assign the A and B compartment, whereby regions with positive PC1 values corresponded to the A compartment and negative values corresponded to the B compartment based on their association with gene density. Loop domains were identified at 10-kb resolution using chromosight^55^.

### CUT&Tag

The CUT&Tag experiments were performed following the CUT&Tag protocol^56^. For each targeted protein, 100,000 cells were used. The following primary antibodies were used at 1:50 dilution: H3K27ac (Cell Signaling Technologies, #8173) and rabbit IgG (Cell Signaling, #2729). We used guinea pig anti-rabbit IgG (H+L) secondary antibody (NBP1–72763) with 1:50 dilution. pA–Tn5 was bought from EpiCypher (15–1117). Final libraries were sequenced as 150-bp paired-end reads on a platform Novaseq X Plus, with raw sequencing depths of 6 million reads.

### CUT&Tag data analysis

CUT&Tag sequencing reads were processed using ENCODE ChIP-seq pipeline. Specifically, reads were first trimmed by Trim_Galore! using the “--paired” option and then aligned to GRCm39 with Bowtie2 (v.2.3.5.1). PCR duplicates were removed using the Picard MarkDuplicates tool with “VALIDATION_STRINGENCY=LENIENT” option. MACS2 (v.2.2.4) was used for peak calling for histone marks and TFs with “-p 1e-2 --nomodel --shift 0 --keep-dup all -B --SPMR” options. Peaks in the ENCODE hg38 blacklist regions (http://mitra.stanford.edu/kundaje/akundaje/release/blacklists/hg38-human/hg38.blacklist.bed.gz ) were filtered out. Peaks with MACS2-reported q value < 10^–5^ were retained for downstream analyses. The output was visualized using chipseq and Gviz in R^57,58^.

### Western blotting

Cells were lysed in Laemmli sample buffer (Sigma-Aldrich) supplemented with protease inhibitor cocktail (Roche Diagnostics). For heart protein, Whole hearts, were lysed on ice for 15 minutes in 120 mM NaCl, 50 mM Tris-HCl (pH 8.0), 1% Triton X-100, protease inhibitor Complete (Roche Applied Science), and phosphatase inhibitors (50 mM sodium fluoride, 1 mM sodium orthovanadate, and 10 mM sodium pyrophosphate). Lysates were cleared by centrifugation at 13000 rpm for 15 minutes at 4°C. Protein concentration was determined by Bradford method. Proteins from hearts or cellular lysates were separated on SDS–PAGE gels (Bio-Rad) and transferred to nitrocellulose membranes (Bio-Rad) for protein detection as described. Antibody used included were: SREBP1 (ABclonal, A26708), PI3K p110α (Cell Signaling Technologies, #4249), p-Akt S473 (Abcam, ab66138), p-Akt T308 (Cell Signaling Technologies, #4056), p-mTOR S2448 (ABclonal, Ap0115), p-mTOR S2481 (ABclonal, AP0978), GSK-3α/ß (Cell Signaling Technologies, #5676), p-GSK-3ß S9 (Cell Signaling Technologies, #9336), β-actin (Santa Cruz Biotechnologies, sc-47778).

### Flow cytometry

#### *Human cells*.

All from Biolegend unless specifically indicated. Fluorescence conjugates targeting human CD33, CD34, CD38, CD117, CD45, CD123, and AT2R (R&D MAB3659) were used. PI or 7-AAD were used for live- and dead-cell discrimination.

#### *Mouse cells*.

All from Biolegend unless specifically indicated: CD3e, Ly-6G/Ly-6C (Gr-1), CD11b, CD45R, CD117 (c-Kit), Sca-1, Ki67, Anti-CD127(IL-7Rα), CD16/32, CD34, PI.

### Virus construction and infection

pLentiLoxp3.7 (addgene #11795) was used to construct *Agtr2* shRNA expressing lentiviral vectors. The target sequences for sh-*Agtr2*-1, sh-*Agtr2*-2 and sh-*Agtr2*-3 were 5’-GCGCCTTTAATTGCTCACACAA-3’, 5’-GCGCCTTTAATTGCTCACACAA-3', and 5’-GCTCACACAAACCATCAGATA-3’, respectively. For virus packaging, retroviral constructs MSCV-MLL-AF9-IRES-GFP or MSCV-AML1-ETO9a-IRES-GFP, were mixed with PCL-ECO (2:1), and the lentivirus constructs were mixed with pSPAX2 and pMD2.G (4:3:1), followed by transfection into 293T cells using PolyJet (SignaGen Laboratories). Virus-containing supernatant was collected 48–72 h post-transfection and used for infection. Briefly, the virus supernatant was collected and filtered through a 0.45-mm filter. Lin^−^ cells were isolated from the BM of littermate *Agtr2*^o/e^ or *Agtr2*^+/+^ mice pretreated with 5-FU (150mg/kg). For infection of Lin^−^ cells from BM, Lin^−^ cells were suspended in virus supernatant supplemented with 4 μg/ml polybrene at 10^5^/ml and placed in six-well plates for spin infection (2000 rpm, 90 min at 32 °C).

### Statistical analysis

Statistical analysis was performed using R. For comparison of two groups, we used Student’s t-test or Wilcoxon rank-sum test and when comparing 3 or more groups, one-way or two-way ANOVA was used with Fisher’s test for post-hoc analyses. The survival rates of the two groups were analyzed using a log–rank test and were considered statistically significant if *P* < 0.05.

## Supplementary Material

Supplement 1

## Figures and Tables

**Fig. 1:**
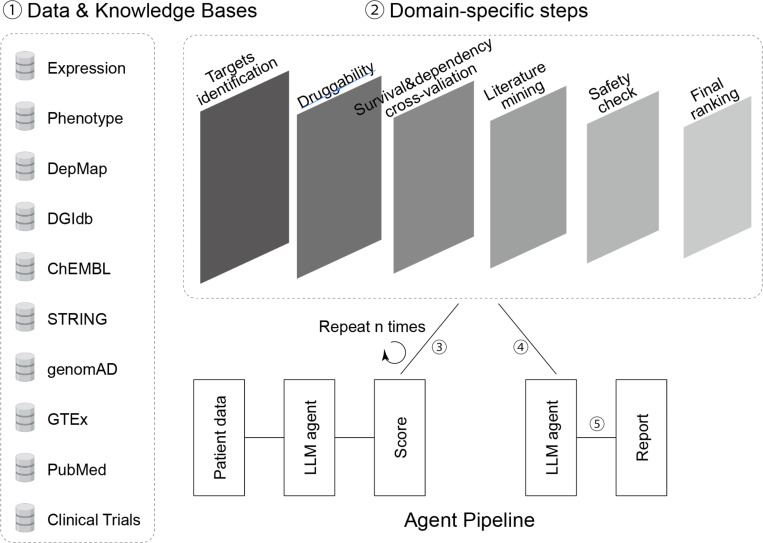
Workflow of the LLM agent for AML therapeutic target nomination The agent integrates multi-modal knowledge bases (expression, phenotype, DepMap, DGIdb, ChEMBL, STRING, gnomAD, GTEx, PubMed, clinical trials) with a structured sequence of domain-specific steps. Starting from RNA-expression and phenotype data in AML cohorts, the pipeline identifies candidate genes associated with favorable ELN 2022 risk groups and therapeutic responses. Targets are filtered for druggability (cell-surface proteins) and assembled into per-gene dossiers incorporating pharmacology evidence (DGIdb, ChEMBL), interaction context (STRING), survival cross-validation (elastic-net Cox using BEATAML), and functional dependency (DepMap). Pathway enrichment analysis (Hallmarks, Reactome) ensures biological coherence. A literature-mining step generates structured summaries of oncogenic role, therapeutic context, novelty, and key references. Human-genetics safety triage applies gnomAD constraint metrics (pLI/LOEUF), GTEx tissue expression, and eQTL breadth to penalize broadly expressed or high-toxicity risk genes. A composite priority score with safety penalty yields a ranked shortlist of targets. Final outputs include a prioritized gene list and transparent, traceable target dossiers highlighting both well-established and understudied AML targets for experimental validation.
