# WITHDRAWN: A Druggable Tumor Suppressor and Leukemic Stem Cell Marker

**DOI:** 10.1101/2025.10.16.682831

**Published:** 2026-03-11

**Authors:** Yi Pan, Xiaduo Meng, Chen Wang, Wenxuan Zhou, Yibo Chen, Richard D. Hammer, Hong Zheng, Gerhard Hildebrandt, Xunlei Kang

## Abstract

The authors have withdrawn this preprint because the co-authors were not in agreement about posting the work in its current form. The authors request that this work not be cited. Please contact the corresponding author (Dr. Xunlei Kang, kangxu@health.missouri.edu ) with questions.

