# Supplementary material for: A Druggable Tumor Suppressor and Leukemic Stem Cell Marker
Source: bioRxiv. 2025 Dec 22:2025.10.16.682831. Preprint. [Version 4] doi: 10.1101/2025.10.16.682831 (PMC12767310; doi:10.1101/2025.10.16.682831)
Supplement: Supplementary file 1 [file NIHPP2025.10.16.682831v4-supplement-1.pdf]

bioRxiv preprint doi: https://doi.org/10.1101/2025.10.16.682831; this version posted December 22, 2025. The copyright holder for this preprint (which was not certified by peer review) is the author/funder, who has granted bioRxiv a license to display the preprint in perpetuity. It is made available under aCC-BY 4.0 International license.

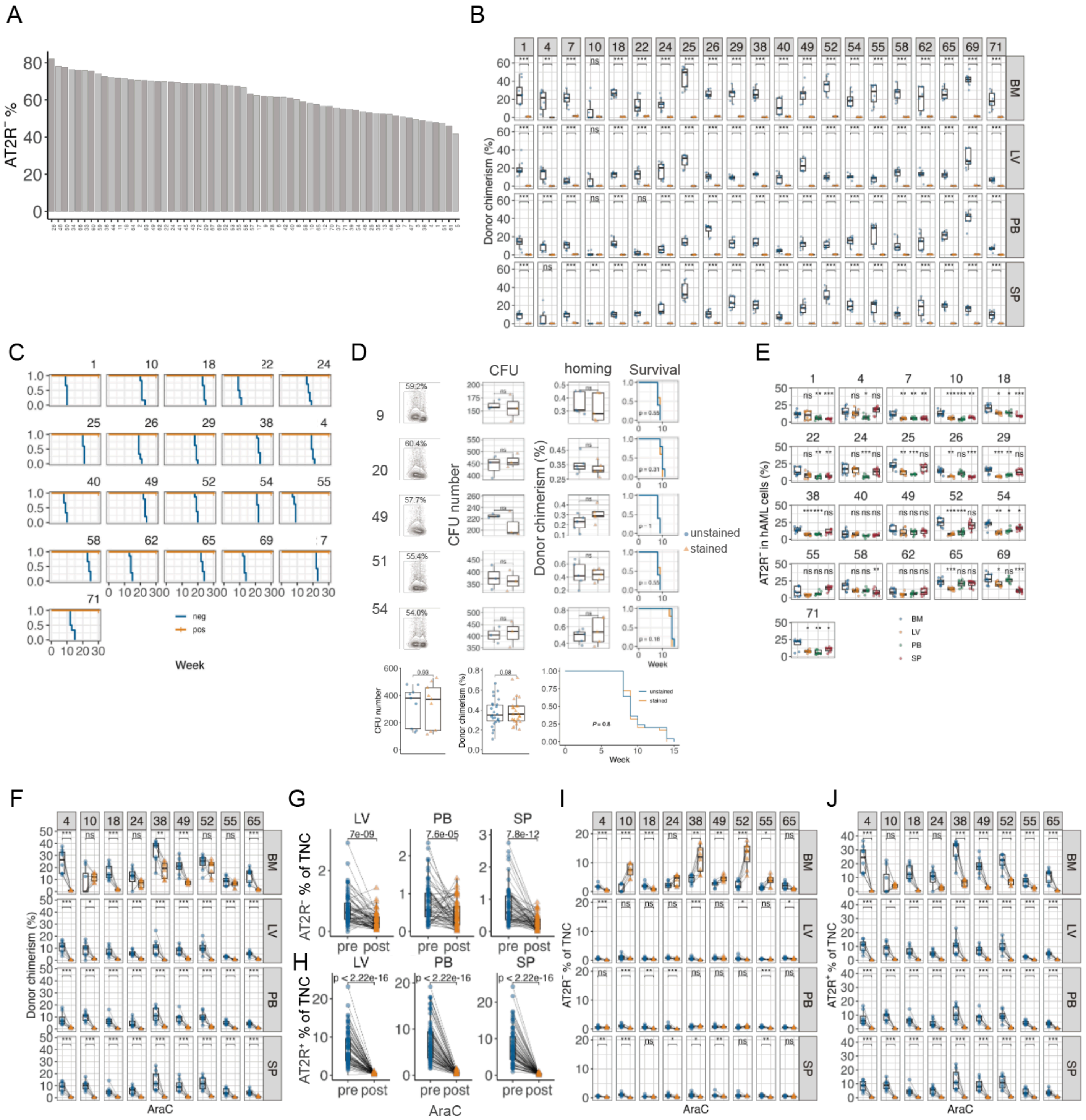

Fig. S1

A, Flow cytometry analysis showing percentages of AT2R$^-$ AML cells in 62 cases of AML.

B, Flow cytometry analysis of long-term engraftment in NSG mice for each human AML sample (n = 8 mice).

C, Survival curve of human AML-transplanted mice for each human AML sample (No. 1 AT2R$^-$, n = 6; No. 1 AT2R$^+$, n = 5; No. 4 AT2R$^-$, n = 6; No. 4 AT2R$^+$, n = 5; No. 7 AT2R$^-$, n = 7; No. 7 AT2R$^+$, n = 5; No. 10 AT2R$^-$, n = 6; No. 10 AT2R$^+$, n = 5; No. 18 AT2R$^-$, n = 6; No. 18 AT2R$^+$, n = 5; No. 22 AT2R$^-$, n = 8; No. 22 AT2R$^+$, n = 5; No. 24 AT2R$^-$, n = 7; No. 24 AT2R$^+$, n = 5; No. 25 AT2R$^-$, n = 5; No. 25 AT2R$^+$, n = 5; No. 26 AT2R$^-$, n = 6; No. 26 AT2R$^+$, n = 5; No. 29 AT2R$^-$, n = 8; No. 29 AT2R$^+$, n = 5; No. 38 AT2R$^-$, n = 8; No. 38 AT2R$^+$, n = 5; No. 40

bioRxiv preprint doi: https://doi.org/10.1101/2025.10.16.682831; this version posted December 22, 2025. The copyright holder for this preprint (which was not certified by peer review) is the author/funder, who has granted bioRxiv a license to display the preprint in perpetuity. It is made available under aCC-BY 4.0 International license.

$AT2R^-$, n = 6; No. 40 $AT2R^+$, n = 5; No. 49 $AT2R^-$, n = 5; No. 49 $AT2R^+$, n = 5; No. 52 $AT2R^-$, n = 8; No. 52 $AT2R^+$, n = 5; No. 54 $AT2R^-$, n = 5; No. 54 $AT2R^+$, n = 5; No. 55 $AT2R^-$, n = 8; No. 55 $AT2R^+$, n = 5; No. 58 $AT2R^-$, n = 6; No. 58 $AT2R^+$, n = 5; No. 62 $AT2R^-$, n = 7; No. 62 $AT2R^+$, n = 5; No. 65 $AT2R^-$, n = 6; No. 65 $AT2R^+$, n = 5; No. 69 $AT2R^-$, n = 7; No. 69 $AT2R^+$, n = 5; No. 71 $AT2R^-$, n = 6; No. 71 $AT2R^+$, n = 5). Log-rank test.

D, AML cells were stained with anti-AT2R antibody or empty control and underwent in vivo CFU assay or in vivo transplantation (CFU, n = 3; homing, n = 5; survival, n = 5). Each dot represents one replicate in CFU and one mouse in in vivo assays.

E, Quantification of $AT2R^-$ AML cells from engrafted mice (post-transplantation) for each human AML sample (n = 8 mice).

F, Mice engrafted with AML cells were treated with AraC (50 mg/kg daily for 5 days) and percentages of AML cells were analyzed before and after treatment for each human AML sample (n = 8 mice). Connected dots depict percentages before and after treatment in one mouse.

G and H, Percentage of $AT2R^-$ and $AT2R^+$ AML cells of total nucleated cells (TNC) were analyzed before and after AraC treatment (n = 9 cases of AML: 4, 10, 18, 24, 38, 49, 52, 55, 65. n = 8 mice). Connected dots depict percentages before and after treatment in one mouse.

I and J, Percentage of $AT2R^-$ and $AT2R^+$ AML cells of total nucleated cells (TNC) for each human AML sample were analyzed before and after AraC treatment (n = 8 mice). Connected dots depict percentages before and after treatment in one mouse.

Unless noted, each dot represents an individual mouse, horizontal bars and whiskers denote median with IQR, and *P* values are annotated and calculated using student's t test (ns, not significant; *$P < 0.01$; **$P < 0.01$; ***$P < 0.001$; ****$P < 0.0001$).

bioRxiv preprint doi: https://doi.org/10.1101/2025.10.16.682831; this version posted December 22, 2025. The copyright holder for this preprint (which was not certified by peer review) is the author/funder, who has granted bioRxiv a license to display the preprint in perpetuity. It is made available under aCC-BY 4.0 International license.

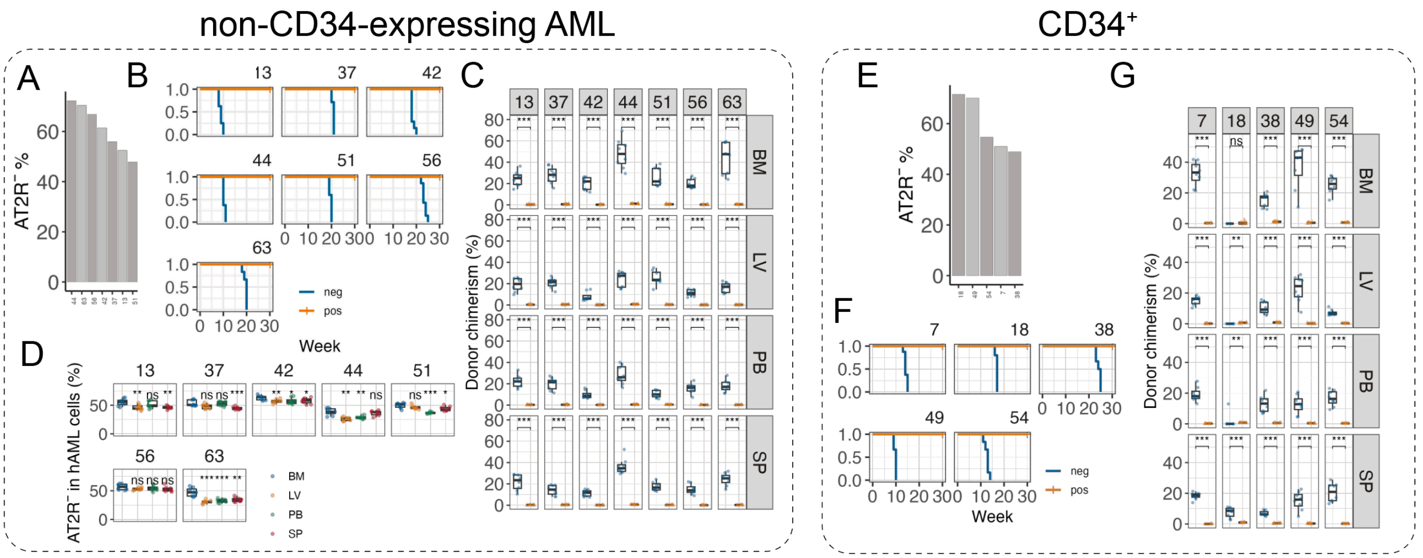

Fig. S2

A, Flow cytometry analysis showing percentages of AT2R⁻ AML cells in 7 cases of non-CD34-expressing AML.

B, Survival curve of human AML-transplanted mice for each non-CD34-expressing human AML sample (Transplanted mice: No. 13 AT2R⁻, n = 8; No. 13 AT2R⁺, n = 5; No. 37 AT2R⁻, n = 7; No. 37 AT2R⁺, n = 5; No. 42 AT2R⁻, n = 7; No. 42 AT2R⁺, n = 5; No. 44 AT2R⁻, n = 8; No. 44 AT2R⁺, n = 5; No. 51 AT2R⁻, n = 5; No. 51 AT2R⁺, n = 5; No. 56 AT2R⁻, n = 7; No. 56 AT2R⁺, n = 5; No. 63 AT2R⁻, n = 6; No. 63 AT2R⁺, n = 5). Log-rank test.

C, Flow cytometry analysis of long-term engraftment in NSG mice for each non-CD34-expressing human AML sample (n = 8 mice).

D, Quantification of AT2R⁻ AML cells from engrafted mice (post-transplantation) for each non-CD34-expressing human AML sample (n = 8 mice).

E, Flow cytometry analysis showing percentages of AT2R⁻ AML cells in 5 cases of high-CD34-expressing AML.

F, Survival curve of human AML-transplanted mice for each high-CD34-expressing human AML sample (Transplanted mice: No. 7 CD34⁺AT2R⁻, n = 8; No. 7 CD34⁺AT2R⁺, n = 5; No. 18 CD34⁺AT2R⁻, n = 5; No. 18 CD34⁺AT2R⁺, n = 5; No. 38 CD34⁺AT2R⁻, n = 6; No. 38 CD34⁺AT2R⁺, n = 5; No. 49 CD34⁺AT2R⁻, n = 6; No. 49 CD34⁺AT2R⁺, n = 5; No. 54 CD34⁺AT2R⁻, n = 6; No. 54 CD34⁺AT2R⁺, n = 5). Log-rank test.

G, Flow cytometry analysis of long-term engraftment in NSG mice for each high-CD34-expressing human AML sample (n = 8 mice).

Unless noted, each dot represents an individual mouse, horizontal bars and whiskers denote median with IQR, and *P* values are annotated and calculated using student's t test (ns, not significant; **P* < 0.01; ***P* < 0.01; ****P* < 0.001; *****P* < 0.0001).

bioRxiv preprint doi: https://doi.org/10.1101/2025.10.16.682831; this version posted December 22, 2025. The copyright holder for this preprint (which was not certified by peer review) is the author/funder, who has granted bioRxiv a license to display the preprint in perpetuity. It is made available under aCC-BY 4.0 International license.

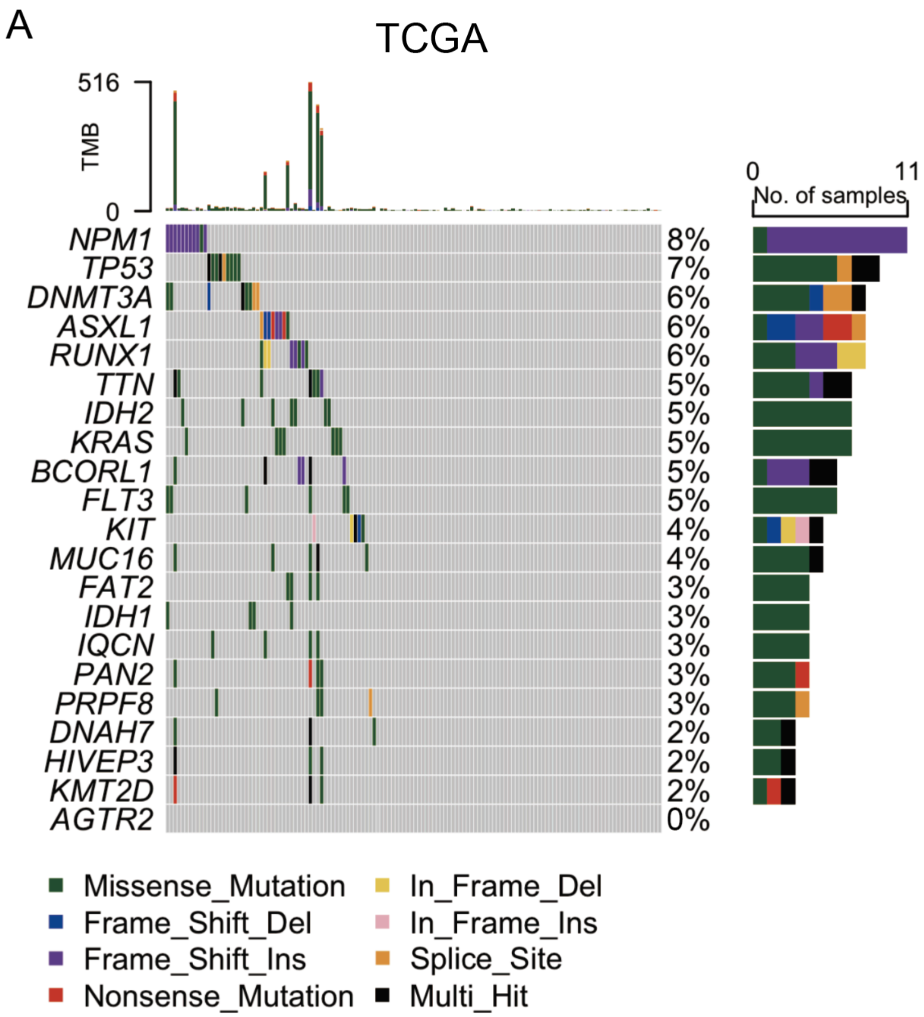

Fig. S3

A, Oncoprint plot showing the distribution and frequency of recurrent somatic mutations across 131 AML patient samples in the TCGA-LAML cohort. Each column represents an individual patient, and each row represents a gene. Top 20 mutated genes and *AGTR2* are shown. Mutation types are color-coded as indicated. The top panel shows tumor mutational burden (TMB) per sample. The bar plot on the right depicts the percentage and number of cases harboring mutations in each gene.

bioRxiv preprint doi: https://doi.org/10.1101/2025.10.16.682831; this version posted December 22, 2025. The copyright holder for this preprint (which was not certified by peer review) is the author/funder, who has granted bioRxiv a license to display the preprint in perpetuity. It is made available under aCC-BY 4.0 International license.

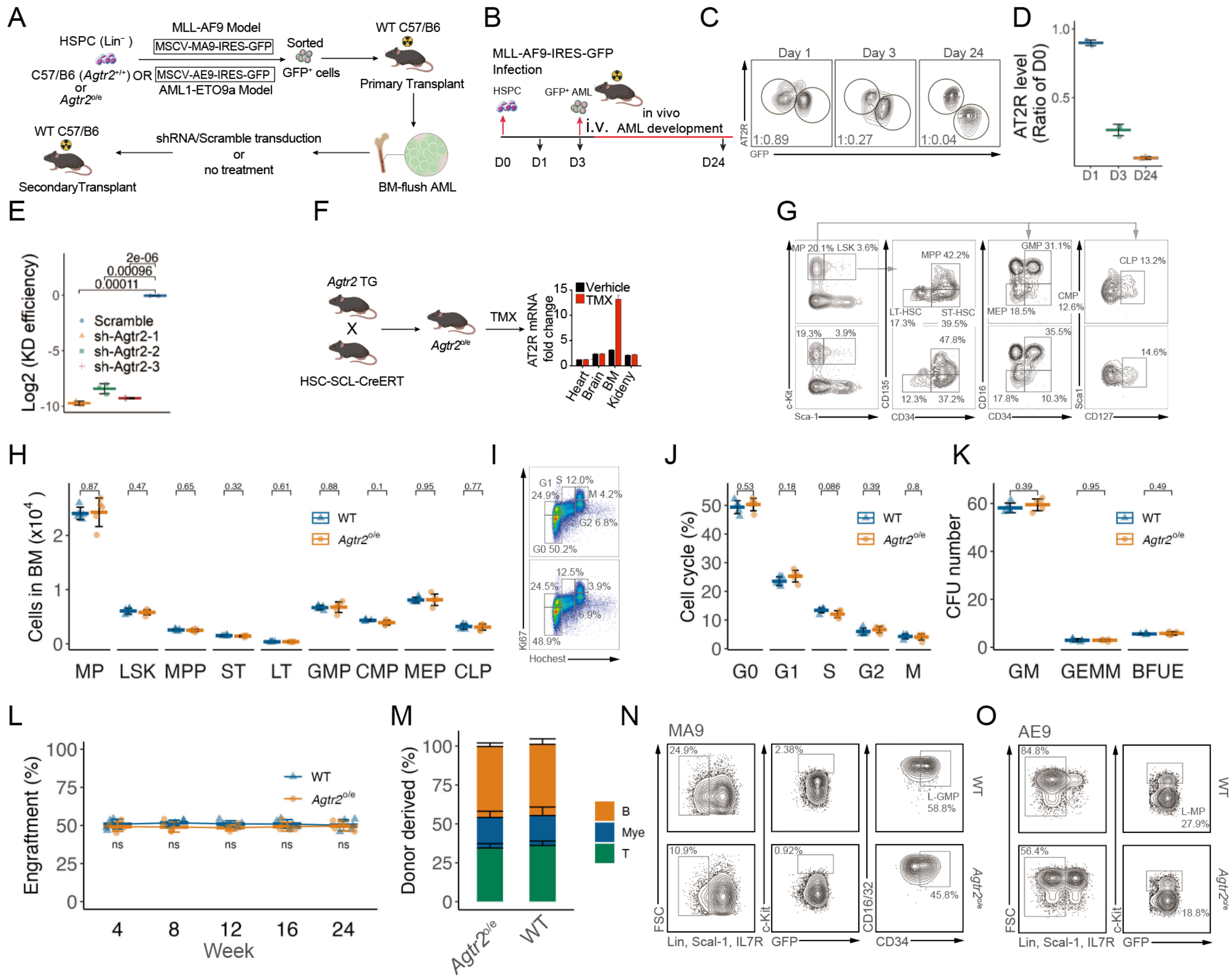

Fig. S4

A, Schematic representation of the generation of the two retrovirus-induced AML models (MLL-AF9 and AML1-ETO9).

B to D, Schematic diagram (B), representative plots (C), and quantification of flow cytometry analysis of AT2R expression on HSPCs 0, 1, 3, and 24 days after MLL-AF9 infection (n=3 mice).

E, KD efficiency of *Agtr2* sh-RNAs (n=3 technical replicates).

F, Schematic diagram of generating inducible hematopoietic system-specific enforced Agtr2 expression.

G and H, Representative flow cytometry plots (G) and quantification (H) of the absolute numbers of HSPC compartments in WT or *Agtr2*$^{o/e}$ mice (n=5 mice). Long-term hematopoietic stem cell, LT-HSC; short-term HSC, ST-HSC; multiple potential progenitors, MPP; Common Myeloid Progenitor, CMP; Granulocyte-Macrophage Progenitor, GMP; Common Lymphoid Progenitor, CLP.

I and J, Representative flow cytometry plots (I) and quantification (J) of the cell cycle stage of BM cells from WT or *Agtr2*$^{o/e}$ mice (n=5 mice).

K, Comparison of BM cell CFUs of WT or *Agtr2*$^{o/e}$ mice (n=3 mice).

bioRxiv preprint doi: https://doi.org/10.1101/2025.10.16.682831; this version posted December 22, 2025. The copyright holder for this preprint (which was not certified by peer review) is the author/funder, who has granted bioRxiv a license to display the preprint in perpetuity. It is made available under aCC-BY 4.0 International license.

L, Competitive reconstitution analysis using 500 HSPCs from WT or *Agtr2*$^{o/e}$ mice (n=5 mice). PB analysis for total engrafted donor cells was plotted against the number of weeks after primary transplantation.

M, Percentages of donor-derived B, T, and myeloid lineage cells from WT or *Agtr2*$^{o/e}$ mice (n=5 mice).

N and O, Gating strategy of identifying LSC-enriched population in MLL-AF9 (L-GMP, N) and AML1-ETO9a (L-MP, O).

Unless noted, each dot represents an individual mouse, horizontal bars and whiskers denote mean and SD, and *P* values are annotated and calculated using student's t test.

bioRxiv preprint doi: https://doi.org/10.1101/2025.10.16.682831; this version posted December 22, 2025. The copyright holder for this preprint (which was not certified by peer review) is the author/funder, who has granted bioRxiv a license to display the preprint in perpetuity. It is made available under aCC-BY 4.0 International license.

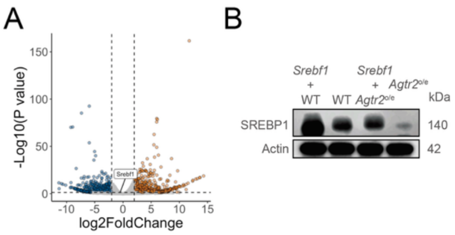

Fig. S5

A, Volcano plot of RNA-seq showing expressing change of *Srebf1* in *Agtr2*$^{o/e}$ compared with MA9 AML cells.

B, Western blot shows SREBP1 expression after *Srebf1* or Ctrl introduction in WT and *Agtr2*$^{o/e}$ MLL- AF9 AML cells.

bioRxiv preprint doi: https://doi.org/10.1101/2025.10.16.682831; this version posted December 22, 2025. The copyright holder for this preprint (which was not certified by peer review) is the author/funder, who has granted bioRxiv a license to display the preprint in perpetuity. It is made available under aCC-BY 4.0 International license.

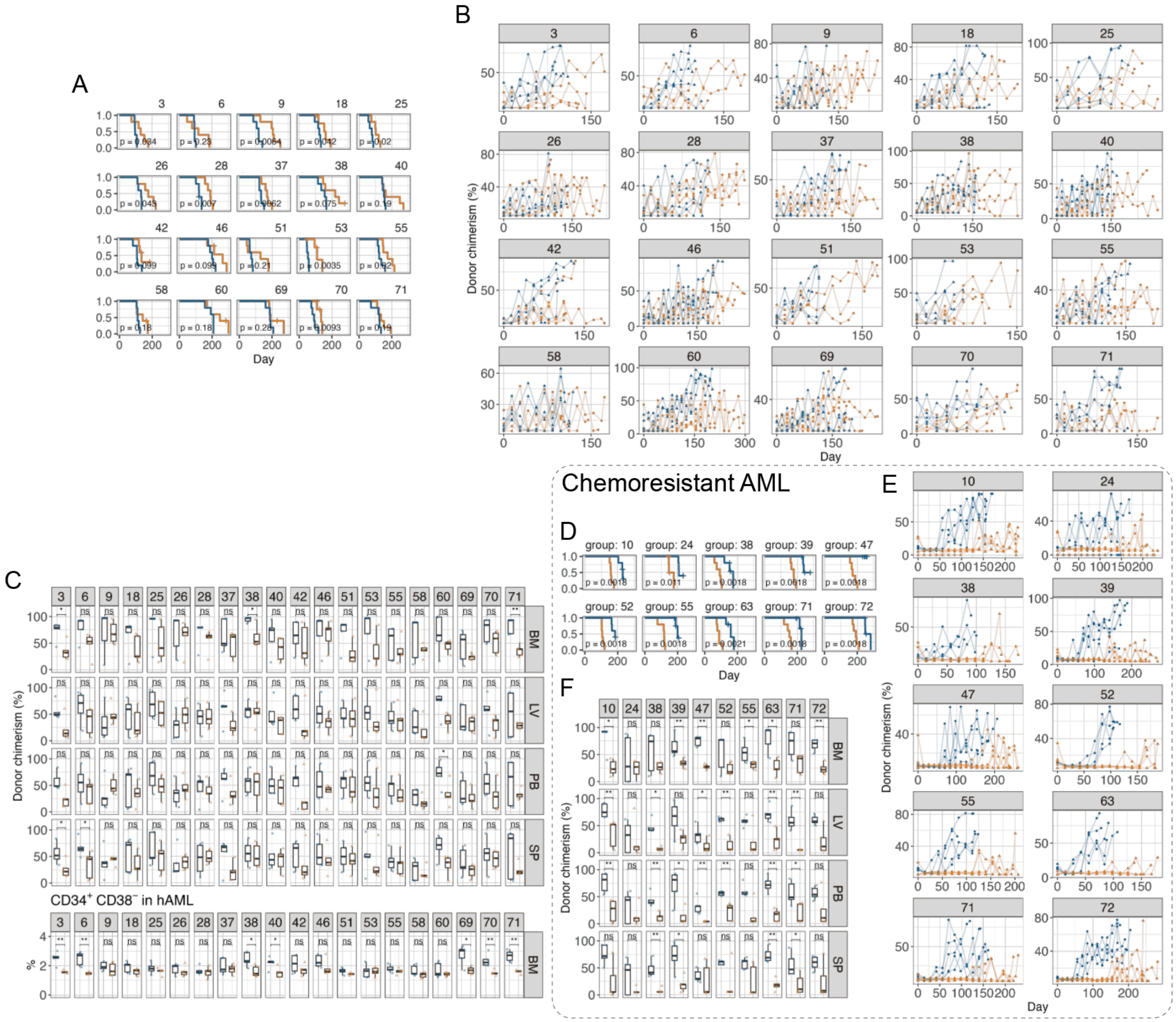

Fig. S6

A, Survival analysis of vehicle control or C21-treated AML PDX models engrafted by each AML sample (n = 5 mice). Log-rank test.

B and C, Peripheral blood (PB) donor chimerism area under the curve (AUC) per day (B) and end point hematopoietic organ donor chimerism and AML surface marker expression of CD34 and CD38 (C) of each AML sample (n = 5 mice).

D, Survival analysis of vehicle control or C21-treated AML PDX models engrafted by each chemo-resistant AML sample (n = 5 mice). Log-rank test.

B and C, Peripheral blood (PB) donor chimerism area under the curve (AUC) per day (B) and end point hematopoietic organ donor chimerism and AML surface marker expression of CD34 and CD38 (C) of each chemo-resistant AML sample (n = 5 mice).

bioRxiv preprint doi: https://doi.org/10.1101/2025.10.16.682831; this version posted December 22, 2025. The copyright holder for this preprint (which was not certified by peer review) is the author/funder, who has granted bioRxiv a license to display the preprint in perpetuity. It is made available under aCC-BY 4.0 International license.

Unless noted, each dot represents an individual mouse, horizontal bars and whiskers denote mean with IQR, and *P* values are annotated and calculated using student's t test.
